# Small molecule modulators of chromatin remodeling: from neurodevelopment to neurodegeneration

**DOI:** 10.1186/s13578-023-00953-4

**Published:** 2023-01-16

**Authors:** Dongfang Jiang, Tingting Li, Caixia Guo, Tie-Shan Tang, Hongmei Liu

**Affiliations:** 1 State Key Laboratory of Membrane Biology, Institute of Zoology, Chinese Academy of Sciences, Beijing, 100101 China; 2 Beijing Institute of Genomics, Chinese Academy of Sciences/China National Center for Bioinformation, Beijing, 100101 China; 3 Beijing Institute for Stem Cell and Regenerative Medicine, Beijing, 100101 China; 4 Chinese Academy of Sciences, University of Chinese Academy of Sciences, Beijing, 100101 China

**Keywords:** Chromatin remodeling, Small molecules, Epigenomics, Histone modifications, Neurodevelopment, Neurodegenerative diseases

## Abstract

The dynamic changes in chromatin conformation alter the organization and structure of the genome and further regulate gene transcription. Basically, the chromatin structure is controlled by reversible, enzyme-catalyzed covalent modifications to chromatin components and by noncovalent ATP-dependent modifications via chromatin remodeling complexes, including switch/sucrose nonfermentable (SWI/SNF), inositol-requiring 80 (INO80), imitation switch (ISWI) and chromodomain-helicase DNA-binding protein (CHD) complexes. Recent studies have shown that chromatin remodeling is essential in different stages of postnatal and adult neurogenesis. Chromatin deregulation, which leads to defects in epigenetic gene regulation and further pathological gene expression programs, often causes a wide range of pathologies. This review first gives an overview of the regulatory mechanisms of chromatin remodeling. We then focus mainly on discussing the physiological functions of chromatin remodeling, particularly histone and DNA modifications and the four classes of ATP-dependent chromatin-remodeling enzymes, in the central and peripheral nervous systems under healthy and pathological conditions, that is, in neurodegenerative disorders. Finally, we provide an update on the development of potent and selective small molecule modulators targeting various chromatin-modifying proteins commonly associated with neurodegenerative diseases and their potential clinical applications.

## Introduction

The nucleosome, consisting of 147 bp of double helix DNA wound around a histone octamer core that possesses one (H3-H4)_2_ heterotetramer and two H2A-H2B heterodimers, is the basic repeating unit of chromatin. They are further packaged into higher-order chromatin structures. The location and structure of nucleosomes affect the function of chromatin. In most eukaryotic cells, chromatin exists in a highly compacted form, which is referred to as heterochromatin. This highly folded chromatin structure is necessary for its packing into the nucleus but limits access of gene promoter regions to the binding of various proteins, such as transcriptional machinery. Chromatin may also adopt a more relaxed state, referred to as euchromatin, which is open to modifications and transcriptional processes. Chromatin remodeling involves a dynamic interchange of chromatin between a condensed state and a transcriptionally accessible state, allowing the regulatory transcription machinery proteins to access condensed genomic DNA and control gene expression. Therefore, it is a vital process to regulate important physiological functions and maintain cellular homeostasis, and impairment of chromatin remodeling machinery leads to the progression of various diseases due to accumulated epigenetic abnormalities.

Eukaryotes have evolved a large family of chromatin remodeling enzymes and related protein factors that alter the location and structure of chromatin. These various chromatin regulatory factors are thus critical determinants of access to genomic loci by the transcriptional machinery, and they function diversely in normal tissues and disease contexts. Consequently, chromatin remodeling is essential for the basal maintenance of the structural homeostasis of chromatin as well as for establishing gene expression patterns [1, 2]. In addition, accumulating evidence strongly suggests that dysfunction of chromatin remodelers contributes to neurodegenerative diseases. In this review, we discuss the epigenetic mechanisms of chromatin remodeling and evidence of how the aberrant functions of each chromatin-modifying protein during neurogenesis and neural development contribute to neurodegenerative disorders. Furthermore, we endeavor to provide an updated description of the use of small molecules as modulators of chromatin remodeling in clinical trials.

## Regulatory mechanisms of chromatin remodeling

**Fig. 1:**
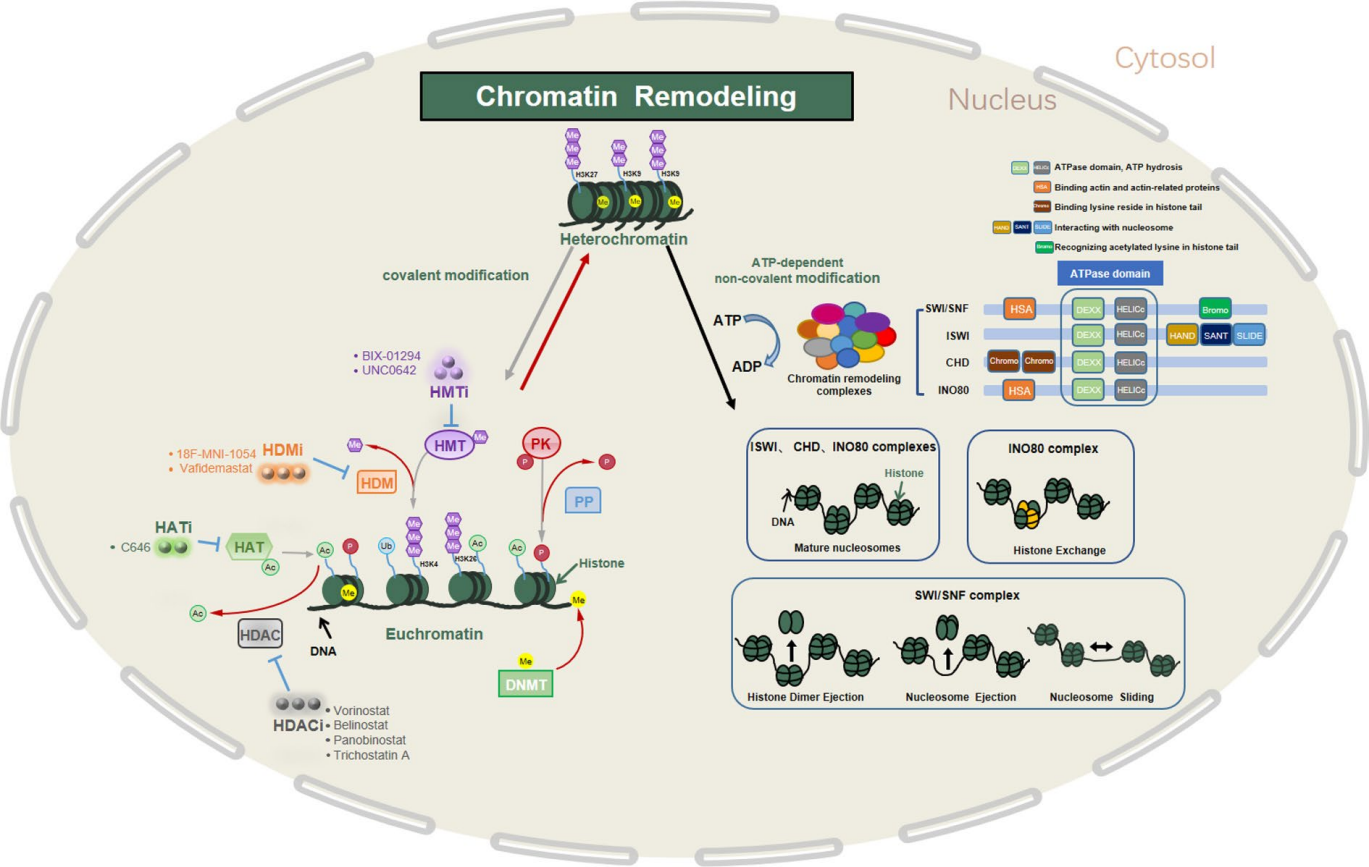
Summary of regulatory mechanisms and the small-molecule drug regulation of chromatin remodeling. Chromatin remodeling involves a dynamic interchange of chromatin between a condensed state (heterochromatin) and a more relaxed state (euchromatin). The underlying mechanisms can be divided into two categories: (1) covalent modification of chromatin components. Epigenetic modifications to histone proteins include but are not limited to acetylation, methylation, phosphorylation and ubiquitination. Enzymes involved include histone acetyltransferase (HAT), histone deacetylase (HDAC), histone methyltransferase (HMT), histone demethylase (HDM), phosphokinase (PK), and phosphatase (PP); DNA methylation is controlled by DNA methyltransferase (DNMT) and DNA demethylase. (2) noncovalent modifications by ATP-dependent CRCs, including the SWI/SNF, ISWI, CHD, and INO80 families. The domain structures of each subfamily of chromatin remodelers are shown. They all contain a conserved ATPase domain, which consists of DEXDc and HELICc domains, thus allowing them to use the energy released from ATP hydrolysis to reposition nucleosomes. Each subfamily member has distinct auxiliary domains and exhibits different modes of nucleosome remodeling (adapted from [46], as detailed in the main text). In addition, some representative small-molecule inhibitors of chromatin remodeling factors are listed. Most of them have been approved for cancer treatment, but in recent years, they have also entered the clinical stage to treat neurodegenerative diseases, as detailed in the last section

A better understanding of the mechanisms of chromatin remodeling will foster the development of new drug targets for improved pharmaceutical interventions. Basically, the mechanisms underlying chromatin remodeling can be divided into two categories: covalent modification of chromatin components (mainly histone-mediated covalent modifications and DNA modification) and noncovalent modifications of ATP-dependent chromatin remodeling complexes (CRCs) [3–10]. The covalent modifications of chromatin components (histones and DNA) change the spatial interaction of DNA and histones to influence the accessibility of transcription machinery to the genomic locus, while the primary function of remodeling complexes is to noncovalently alter the positioning and structure of nucleosomes by interacting with DNA and histones via several special recognition domains (Fig. 1). These mechanisms function individually and in concert to regulate gene expression, as detailed next. In addition, noncoding RNAs and histone mutations have also been shown to have an important influence on chromatin structure and transcription (see review [11]), which we will not go further here.

## Covalent modification of chromatin components

Histones, predominantly globular proteins with flexible N- and C-terminal tails, are once considered to be static and nonparticipating structural elements, but the bias is clarified as research reveals that histones are dynamic regulators responsible for gene transcription. The electrostatic interactions between the positively charged histone tails and negatively charged DNA make chromatin in a highly condensed state. Diverse posttranslational modifications (PTMs) occur in the tail region of histones. Epigenetic modifications to histone proteins, including but not limited to acetylation, methylation, phosphorylation, ubiquitination, sumoylation, crotonylation and glycosylation, influence transcriptional access to genes by changing chromatin structure or recruiting histone modifiers. When histones are modified in any way mentioned above, they endure changes in charge characteristics and binding sites [12]. Currently, at least 80 different types of histone modifications have been identified, with acetylation and methylation being the most extensively studied. Histone modifications can be gene-specific within the genome and site-specific within a given chromatin particle. Importantly, site-specific histone modifications directly change chromatin state and transcription. Some modifications, such as histone acetylation or phosphorylation, alone or in a specific combination, are generally associated with transcriptional activation, while others are correlated with transcriptional repression.

Histone acetylation is one of the most common and best-studied histone PTMs and involves the addition of an acetyl group to the lysine residues at the amino-terminal tails of histone proteins. The balance between acetylation and deacetylation of histones is reversibly controlled by histone acetyltransferase (HAT) and histone deacetylase (HDAC). Generally, histone acetylation mediated by HATs leads to the relaxation of chromatin structure and facilitates gene activation, whereas deacetylation of lysine residues on histone tails mediated by HDACs results in a more closed chromatin structure and represses gene expression. However, both HATs and HDACs have been found to exist in some large protein complexes, suggesting that a great deal of fine-tuning occurs at the level of DNA and the addition and subtraction of acetyl groups. Most HATs function as transcriptional coactivators. In humans, there are approximately 30 known HATs, which have been classified into two general categories based on their subcellular localization: Type A and Type B. Type A HATs, which contain a bromodomain, are predominantly localized in the nucleus and acetylate nucleosomal histones and other chromatin-associated proteins. Type B HATs, which lack a bromodomain, are mostly found in the cytoplasm and acetylate free newly synthesized histones in the cytoplasm prior to incorporation into chromatin. In mammals, the HDAC family consists of 18 members that can be divided into four classes (Table 1). The 11 HDACs from classes I, II, and IV are metal-dependent enzymes and require a divalent metal ion for catalysis, while class III sirtuin HDACs are nicotinamide adenine dinucleotide (NAD)-dependent enzymes with protein deacetylase and ADP-ribosylase activity.

**Table 1:** The classification, structure, location and identified substrates of HDACs

Class	Member	Homology to yeast	Structure	Location	Deacetylase activity	Identified/high-confidence substrates
Zn^2+^ -dependent HDACs:						
Class I	HDAC1	Yeast RPD3		N	Strong deacetylase activity only to form Sin3/NuRD/CoREST complex	CDK1, AIFM1, MSH6, RuvB-Like1, MEF2, Histones, GATA, KIF11, YY1, NF-ҡB, DNMT1-SHP, ATM, MyoD, p53, AR, BRAC1, MECP2, pRb
	HDAC2			N		CDKN1A, HOP, MDM2, Histones, NF-ҡB, GATA2, BRCA1, pRb, MECP, IRS-1
	HDAC3			N, C	Strong deacetylase activity only to form SMRT/NCoR complex	FoxO3, SHP, GATA1, NF-ҡB, pRb, Histones, HDAC4,5,7–9
	HDAC8			N, C	Strong deacetylase activity alone	ARID1A, ERR-α, SMC3, RAI1, ZRANB2, NCOA3, THRAP3, HSP70, p53
Class IIa	HDAC4	Yeast HDA1		N, C	Strong deacetylase activity only to form SMRT/NCoR-HDAC3 complex	PGC-1α, HSC70, Histones, SRF, Runx2, p53, p21, GATA, FOXO, HIF-1α, SUV39H1, HP1, HDAC3, MEF2, CaM, 14–3-3
	HDAC5					Histones, HDAC3, YY1, MEF2, Runx2, CaM, 14–3-3
	HDAC7			N, C	Strong deacetylase activity only to form SMRT/NCoR-HDAC3 complex	Histones, HDAC3, MEF2, PML, Runx2, CaM, 14–3-3, HIF-1α
	HDAC9					TRIM29, Histones, HDAC3, MEF2, CaM, 14–3-3
Class IIb	HDAC6	Yeast HDA2		N, C	Strong deacetylase activity alone	MYH9, HSC70, DNAJA1, α-tubulin, Cortactin, HSP90, HDAC11, SHP, PP1, Runx2, LcoR
	HDAC10					HSC70, LcoR, PP1
Class IV	HDAC11	Yeast RPD3/HDA1		N	Strong deacetylase activity alone	SHMT2, Histones, HDAC6, Cdt1
NAD + -dependent HDACs:						
Class III	SIRT1	Yeast Sir2		N, C	Strong deacetylase activity	p53, p73, p300, NF-ҡB-FOXO, PTEN, NICD, MEF2, HIFs, SREBP-1c, β-catenin, PGC1α, Bmal, NF-ҡB, Histones, Per2, Ku70, IRS-2, APE1, PCAF, TIP60, AceCS1, PPARγ, ER-α, AR, LXR
	SIRT2			N, C	Strong deacetylase activity	Hsp90α, Histones, α-tubulin
	SIRT3			M	Strong deacetylase activity	IDH2, HMGCS2, GDH, AceCS, SdhA, Histones, Ku70, SOD2, LCAD, ICDH
	SIRT4	Yeast Sir2		M	Weak deacetylase activity	MCD, PDH, GDH
	SIRT5			M	Weak deacetylase activity	Cytochrome c, MAVS, CPS1, ACAT1
	SIRT6			N	Weak deacetylase activity	FoxO1, Histone H3, p53, NAMPT, TNF-α
	SIRT7			NO	Weak deacetylase activity	Histone H3, p53, PAF53, NPM1, GABPβ1

Classical deacetylase domain,  Nuclear localization signal,  Nuclear export signal,  MEF binding motif,  14–3-3 binding site,  Zinc finger,  Leucine-rich domain,  NAD + -dependent deacetylase domain

*N* Nuclear; *C* cytoplasmic; *M* mitochondrial; *NO* Nucleolar

Chromatin structure is also regulated by histone methylation, which is controlled or regulated by histone methyltransferases (HMTs) and histone demethylases (HDMs). Methylation occurs primarily on at least 11 lysine and arginine residues in histones, with the lysine residues being mono-, di- and trimethylated and the arginine residues being mono-, symmetric di- and asymmetric di-methylated. Thereby, HMTs and HDMs not only have a high degree of specificity for the specific lysine/arginine but also strictly regulate the degree of methylation of their substrates and products. HMTs comprise approximately 59 proteins [13]. There are mainly two types of histone lysine demethylases: the amine oxidase demethylases LSD1 (known as KDM1A) and LSD2 (KDM1B) and the Jumonji C domain (JmjC) family, which has approximately 23 members [14]. Histone methylation occurs more frequently in H3 than in H4. Differential histone methylation may either repress or activate gene transcription, depending on the particular actual residue that is methylated and the degree of methylation.

Histone ubiquitination influences chromatin dynamics and exhibits crosstalk with other histone modifications [15, 16]. H2A and H2B are two of the most abundant ubiquitinated proteins in the nucleus. In addition, ubiquitination has also been reported in H3, H4 and the linker histone H1. H2B monoubiquitylation correlates with transcriptional activation and gene silencing and is also essential for homologous recombination through chromatin remodeling by recruiting chromatin remodeling factors. Ubiquitylation of H2A is associated with transcriptional repression.

Histone phosphorylation, which is controlled by kinases and phosphatases, usually takes place on serine, threonine and tyrosine residues. The majority of histone phosphorylation occurs in the N-terminal tails of H3, followed by H2B, H2A, H4 and H1. H3 S10 phosphorylation, which can be intact with H3 acetylation/methylation, is vital for chromosome condensation and transcriptional activation of eukaryotic genes in various organisms. The phosphorylation of histones also affects the level of ubiquitination of H2B at K120 [17].

DNA methylation, the major form of DNA modification, which involves the covalent addition of a methyl group (-CH3) to the 5´ carbon of cytosine residues (5mC) predominantly in the context of CpG dinucleotides, is generally associated with repressed regions of the genome. DNA methylation is mediated by DNA methyltransferases (DNMTs) that catalyze the transfer of the methyl group from S-adenosylmethionine onto cytosine [18]. DNMT1 and DNMT3 have been shown to play pivotal roles in DNA methylation. In mammals, the DNMT3 family members, DNMT3A, DNMT3B, and the DNMT3-like nonenzymatic regulatory factor DNMT3L, are responsible for the de novo establishment of DNA methylation patterns during early embryogenesis [19], while DNMT1, together with the DNMT1 cofactor UHRF1, functions to maintain these patterns during cell divisions [20]. In male germ cells, DNMT3c, which arose through a single duplication of Dnmt3B without the PWWP domain, is found to be a new de novo DNA methylation enzyme that maintains male fertility by methylating and inhibiting transposon activity [21].

DNA methylation carries out distinct functions in different genomic regions. It is associated with gene repression in mammals by attracting or repelling various DNA-binding proteins. DNA methylation can prevent transcription by inhibiting the binding of transcription factors to target sites. On the other hand, it can suppress gene transcription by recruiting repressor complexes to methylated promoter regions. Recently, it is reported that DNA methylation can also induce gene activation when it replaces H3 lysine 27 trimethylation (H3K27me3), a histone modification mediated by the polycomb repressive complex (PRC) and relates to gene silencing [22]. DNA methylation patterns, which can be affected by histone modifications and chromatin remodeling enzymes, can promote heterochromatin formation [23, 24].

DNA demethylation is realized by either an active pathway involving the dioxygenases called ten-eleven translocation (TET) proteins, including TET1, TET2 and TET3, to remove the methyl group from 5mC, or a passive pathway by lack of maintenance methylation (mediated by DNMT1) during cell divisions [25]. In the process of cell-type transition, methylated DNA can also be recognized by transcription activators, such as the cell pluripotency factors KLF4 and OCT4, the homeobox protein HOXB13 and the NKX neural patterning factor [26], which then recruit the dioxygenase TET2 to induce demethylation, leading to cell fate reprogramming [27].

### Noncovalent regulation: ATP-dependent chromatin modifications

In addition to covalent modifications, the chromatin architecture and access can also be modulated by noncovalent chromatin modifications involving ATP-dependent CRCs. CRCs are evolutionarily conserved multisubunit assemblies of ATP-dependent remodeling enzymes. Based on the sequence and structure of the ATPase subunit, CRCs have been grouped into four main families: switch/sucrose nonfermentable (SWI/SNF), inositol-requiring 80 (INO80), imitation switch (ISWI) and chromodomain-helicase DNA-binding protein (CHD), each representing a specific remodeling pathway with distinct subunits and functions [28]. Other uncategorized ATPase remodelers include Cockayne syndrome group B (ERCC6/CSB), alpha thalassemia/mental retardation syndrome X-linked (ATRX) and Rad54L. The four family remodeler complexes share a conserved enzymatic ATPase subunit, allowing them to use the energy released from ATP hydrolysis to reposition nucleosomes. They can regulate the chromatin structure dynamically by sliding, ejecting, dis/assembling, and repositioning the nucleosome as well as exchanging histone variants. However, the precise molecular mechanisms of subunit organization, assembly pathway and nucleosome recognition of CRCs remain largely unknown [29].

Mammalian SWI/SNF (mSWI/SNF) complexes, canonical BRG1/BRM-associated factor (BAF) and polybromo-associated BAF (PBAF) and newly defined noncanonical BAF (ncBAF), are composed of up to 15 core subunits, with an interchangeable core ATPase subunit (BRG1 or BRM) and different BAF subunits [30]. Among them, some subunits are evolutionally conserved, such as BRG1/BRM, BAF155/170, BAF47, BAF53, BAF250a/b, and BAF60, while some are more recently evolved, such as BCL11a/b, BCL7a/b/c, BRD7/9 and SS18/CREST. BAF complexes have intrinsic nucleosome disassembly [31] and sliding activity and unique ejection activity, which create nucleosome-depleted regions that are essential for transcriptional regulation [32, 33]. The polymorphic BAF and PBAF complexes can activate and repress many eukaryotic genes by interacting with certain transcription factors and are essential for mammalian development [2, 34].

The INO80 remodeler has been identified in yeast, flies and mammals and is present as several complexes. The mammalian complexes are INO80, SRCAP (yeast SWRI or SWR-C) and TRAAP/Tip60 (yeast NuA4). Due to the presence of an insertion in the ATPase domain to which the subsequently recruited helicases (Ruvbl1 and Ruvbl2) could bind [29], the catalytic ATPase subunit of INO80 has helicase activity. The INO80 remodeler modifies chromatin in a number of ways. Compared with other chromatin remodeling agents, the INO80/SWR complex does not change the location or occupancy of nucleosomes. Instead, they change classic histones (such as H2A) in nucleosomes to nonclassical variants (e.g., H2A. Z) [35] or adapt standard nucleosomes to include variant histones [36]. The nucleosome remodeling activity directed by INO80 provides accessibility to certain chromatin domains by catalyzing nucleosome sliding during transcription [37]. INO80 also mediates remodeling activities such as nucleosome spacing at genes and phasing [38, 39]. Chromatin remodeling mediated by INO80 can act as both an activator and a repressor to regulate gene transcription [40].

The ISWI complex is first identified in Drosophila with orthologs in yeast and mammalian systems. Similar to other remodelers, ISWI is present as several complexes that are primarily represented by chromatin assembly factor (CAF), chromatin accessibility complex (CHRAC) and nucleosome-remodeling factor (NURF) in humans [41, 42]. Mammals possess two ISWI orthologs, *Smarca5* and *Smarca1* (encoding SNF2H and SNF2L, respectively) [43]. In mammals, SNF2H protein is abundant and widely distributed, while SNF2L is biased toward tissue-specific distribution and has low abundance [44]. The ISWI remodeling factor has two domains, namely, the N-terminal catalytic ATPase domain, which mediates the interaction with DNA, and the C-terminal HAND-SANT-SLIDE (HSS) domain, which is responsible for the interaction with exosome DNA and the H4 histone tail [45]. Generally, ISWI complexes assist in the maturation of prenucleosomes into octameric mature nucleosomes as well as the correct and regular spacing of newly formed nucleosomes [46].

The CHD family of ATPases has N-terminal tandem chromodomains in addition to the conserved DEAD/H ATPase domain. In humans, this family has nine members, which have been subdivided into three subfamilies according to the presence or absence of additional domains. In addition to dual chromodomains and ATPase domains, subfamily I, which includes CHD1 and CHD2, contains a DNA-binding domain that preferentially binds to AT-rich DNA motifs at the C-terminus. Subfamily II, which includes CHD3, CHD4 and CHD5, contains two N-terminal plant-homeodomain (PHD) with histone-binding activity. Subfamily III, which includes CHD6, CHD7, CHD8 and CHD9, contains an SANT domain and a C-terminal Brahma and Kismet (BRK) domain. As CHD5 has both PHD finger and SANT domains, there is a discrepancy in the protein classification of CHD5 [47]. The diverse functional domains of CHD proteins imply their different roles as ATP-dependent chromatin remodelers. In terms of chromatin-remodeling activities, these CHD enzymes exhibit distinct differences. CHD1 plays a key role in nucleosome assembly and can regulate the chromatin structure through nucleosome spacing or sliding. CHD3 and CHD4, core subunits of nucleosome remodeling and deacetylation (NuRD) complexes, have been reported to slide nucleosomes along DNA and to direct nucleosomal sliding toward adjacent free DNA. CHD5 is reported to lack robust nucleosome sliding activity but remodel nucleosomes by unwrapping [48]. For subfamily III CHDs, while both CHD7 and CHD8 can slide nucleosomes, CHD6 disrupts nucleosomes in a largely nonsliding manner [49].

## The physiological functions of chromatin remodeling in the nervous system

### Chromatin remodeling in the central nervous system (CNS) neurodevelopment

**Fig. 2:**
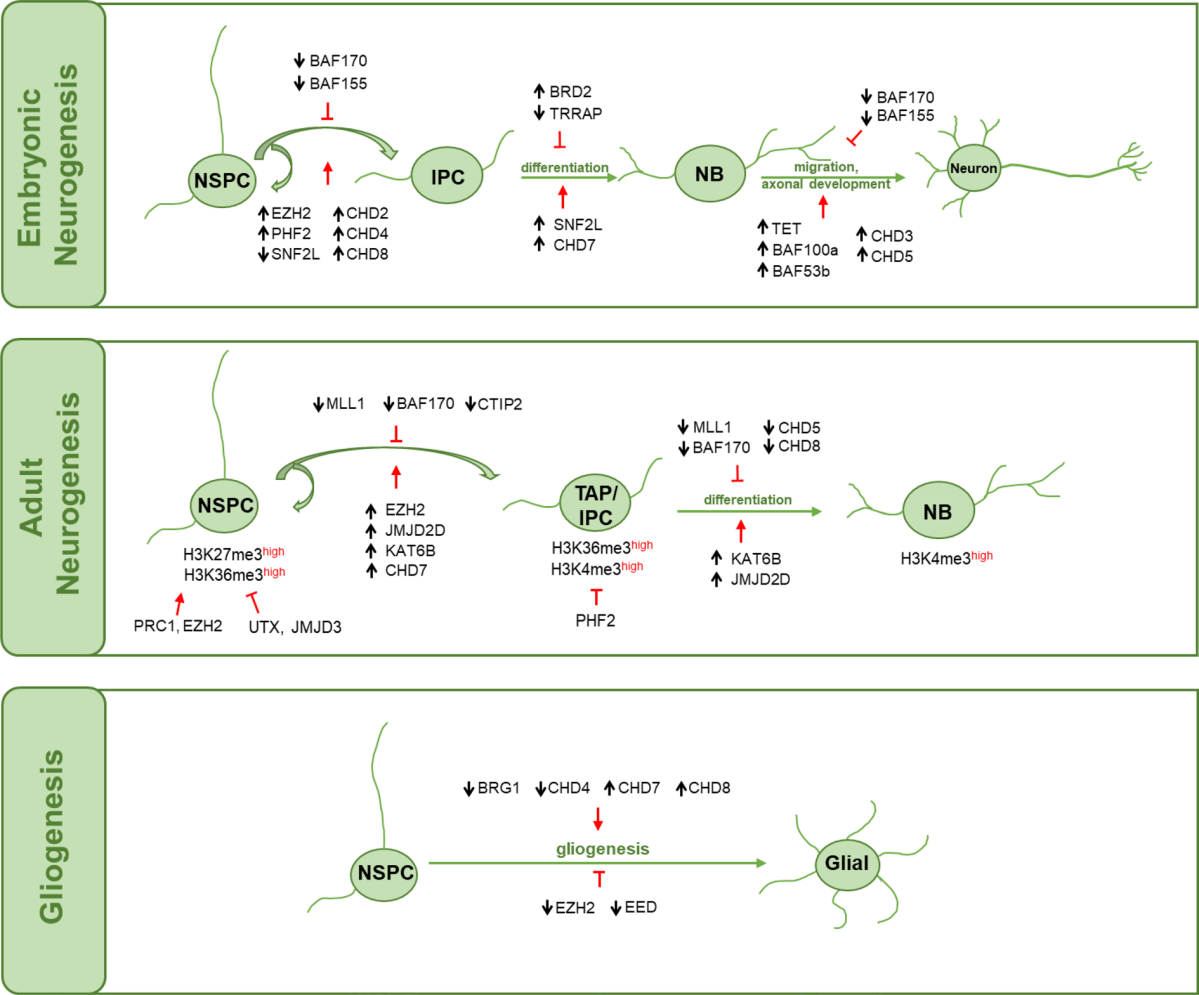
Roles of chromatin remodeling factors in the CNS neurodevelopment. In the CNS, neurodevelopment requires appropriate modulation, such as chromatin remodeling, to ensure that NSPCs can undergo self-renewal, differentiation, migration and ultimate maturation to produce new neurons. Representatively, during the embryonic neurogenesis, some dynamic chromatin remodeling factors would promote the proliferation and renewal of NSPC, such as increased polycomb repressive complexes EZH2, histone demethylase of H3K9me2 PHF2, CHD complexes CHD2, CHD4 and CHD8 and decreased ISWI complex SNF2L. Conversely, the downregulation of SWI/SNF complexes BAF170 and BAF155 would inhibit this process. Further, increased ISWI complex SNF2L, CHD complexes CHD7 would promote the differentiation of IPC to NB, while decreased TRRAP and increased BET proteins BRD2 would block this process. Moreover, increased DNA dioxygenases TET, SWI/SNF complex BAF100a and BAF53b, CHD complexes, including CHD3 and CHD5, would stimulate the migration and maturation of NB to neuron, while decreased SWI/SNF complexes (BAF170 and BAF155) would inhibit that. However, there are diverse CRCs involved in adult neurogenesis. For example, in addition to BAF170 and EZH2, histone acetyltransferase KAT6B, histone lysine demethylase 2 JMJD2D, histone methyltransferase MLL1 and CHD7 also positively regulate the proliferation and renewal of adult NSPC. Further, increased KAT6B and JMJD2D promote the differentiation of TAP to NB, while decreased MLL1, BAF170, CHD5 and CHD8 would block the process. In parallel, the downregulation of SWI/SNF complex BRG1 and CHD complexes, including CHD4, CHD7 and CHD8, would transform the differentiation of NSPC to glial

In the neural development of the early embryo and after birth, neurogenesis is a delicately organized developmental event that requires appropriate modulation of neural stem/progenitor cells (NSPCs) to proliferation, differentiation, migration, and ultimate maturation to neurons and glial cells [50, 51]. In contrast to the widespread embryonic neurogenesis, adult neurogenesis takes place mainly in the subventricular zone (SVZ) of the lateral ventricle and the subgranular zone (SGZ) of the dentate gyrus (DG) of the hippocampus. Some NSCs in the hippocampus persist in the brains of adult mammals and continue to produce new neurons throughout life, and this process is thought to be involved in learning and memory [52]. The whole process of neurogenesis is critically regulated by external and inherent factors, including epigenetic factors [53]. Among such epigenetic programs, chromatin modification, as an indispensable part, causes genome-wide changes in chromatin dynamics during neurogenesis, which corresponds to the guiding gene expression patterns of diverse cell lineages. In this section, we review the involvement of chromatin remodeling proteins in neurogenesis and neurodevelopment by focusing specifically on histone and DNA modifications as well as the four classes of CRCs (Fig. 2).

#### Histone and DNA modifications

One of the most common models of chromatin regulation during neural development is the histone modification “bivalency” [54], that is, histone methylation and acetylation [55]. Dynamic changes in histone status, especially those in the promoter regions, are regarded as the basis of transcriptome dynamics during development. The chromatin profile of neural progenitor cells reinforces the notion that the different states of histone markers affect cell identity. Diverse histone methylations in the promoter regions regulate specific gene transcription activities and cell fate decisions [56]. H3 lysine 4 trimethylation (H3K4me3) and H3 lysine 36 trimethylation (H3K36me3) are usually markers of chromatin regulation concerned with gene activation [57], while H3K27me3 is a marker related to gene silencing [58]. When H3K4me3 and H3K27me3 modifications occur in the same promoter region, they will be considered transcriptionally inactive but “accumulate” for further activation [59]. These histone methylations are reported to play important roles in diverse stages of neurogenesis.

Specifically, during postnatal neurogenesis in the mouse SVZ region, different histone methylation patterns have been observed in different subtypes of NSPCs. Ependymal-like stem NSPCs and quiescent NSPCs (qNSCs) are marked by high levels of H3K27me3, and qNSCs and transient amplifying progenitors (TAPs) are mainly modified by H3K36me3, while type C cells and migrating neuronal precursors (neuroblasts, NBs) are mainly modified by H3K4me3 [60]. These findings reveal how different histone methylation marks are dynamically regulated during NSPC differentiation in the SVZ, thus shedding light on neurodevelopment onset, which supports the essential role of epigenetic regulation in neurogenesis.

The deposition and downstream actions of H3K27 methylation are commonly mediated by PRC, including PRC1 and PRC2. Specifically, PRC2 is responsible for catalyzing and maintaining H3K27me3, while PRC1 is responsible for recognizing H3K27me3 and catalyzing histone H2A ubiquitination modifications, further promoting chromatin aggregation and inhibiting the recruitment of the transcription initiation complex [61]. Loss-of-function mutations in PCR1 components, e.g., the polycomb component Ring1b, or PCR2 components, e.g., EED, or enhancer of Zeste Homologs 2 (EZH2), can cause defects in the conversion of neurogenic cells to astrocytes during embryonic cortical development [62]. Specifically, EZH2, a catalytic subunit of PRC2 with methyltransferase activity, is important for the regulation of embryonic cortical neurogenesis [63] and postnatal birth neurogenesis in the adult SVZ [64] and hippocampus [65]. EZH2 inhibits the expression of GFAP by interacting with CHD4, thus preventing early glial cell generation during neocortex development [66]. In embryos, EZH2 maintains the self-renewal and neural tube apicobasal polarity of neural progenitors by regulating p21^WAF1/CIP1^ protein levels [67], whereas in the postnatal SVZ, EZH2 promotes cell proliferation by inhibiting the Ink4a/Arf locus and confers SVZ lineage specificity by inhibiting non-SVZ neuronal differentiation [64]. In the mouse postnatal brains, the absence of methyltransferase MLL1 not only lowers postnatal neurogenesis but also prevents normal migration of neuronal precursors and their differentiation into neural cells, which is caused by silenced bivalent loci at *Dlx2* (H3K4me3 and H3K27me3) in postnatal neural precursors [68]. These results suggest that different neuronal subtypes require different chromatin-remodeling genes for differentiation.

HDMs have also been shown to be essential in development. LSD1, the first discovered demethylase, demethylates mono- and di-methyl H3K4 via flavin adenosine dinucleotide (FAD)-dependent oxidation to inhibit neuron-specific genes in nonneuronal cells [69]. The Jumonji domain-containing histone lysine demethylase 2 (JMJD2, also known as KDM4) family mediates chronic stress-induced changes in hippocampal neurogenesis by removing H3K9me2, the repressive methylation mark. JMJD2D is found to regulate NSPC differentiation by regulating transcription factors involved in controlling proliferation and differentiation [70]. Moreover, JMJD3 (also known as KDM6B), an H3K27me3 demethylase that counteracts the PRC2 complex [71], can activate neurogenic gene expression via H3K27 demethylation at both promoters and enhancers in NSC populations. Conditional deletion of *Jmjd3* causes neurogenic defects in the SVZ region of postnatal mice [72]. UTX (also known as histone demethylase 6a, KDM6a) is another H3K27me3 demethylase that has been shown to be associated with neurodevelopment. PHD Finger Protein 2 (PHF2), a histone demethylase of H3K9me2, is reported to be essential for the proliferation of neural progenitors, the deletion of which leads to R-loop accumulation, DNA damage, and cell cycle arrest, thus functioning as a guarantor of genome integrity during early embryonic neurogenesis [73].

Increasing evidence has shown that histone acetylation plays an essential role in brain development [74, 75]. KAT6B, also known as MORF, is highly expressed in the region of neurogenesis and can promote the self-renewal of NSPCs [76]. Histone deacetylation has been increasingly recognized for its role in embryonic neural development [77]. Among the HDAC families, class I deacetylases are key drivers of embryonic neurogenesis through the regulation of H3K9ac [78]. They are also found to act extensively in neurogenesis, neural circuit formation and synaptic transmission [79].

While writers (e.g. HATs) and erasers (e.g. HDACs) of histone acetylation have been extensively investigated, the role of histone acetylation readers in brain development has only recently emerged. Bromodomains (BRDs), a diverse family of evolutionarily conserved protein-interaction modules that bind to acetyl-lysine modifications on histone and nonhistone proteins, are essential for the targeting of chromatin-modifying enzymes to specific sites. BRDs are commonly subclassified into bromodomain and extraterminal domain (BET) and non-BET families. Due to their ability to bind to acetylated histones and act as scaffolds for chromatin-modifying complexes, BET proteins function as epigenetic readers of lysine acetylation. BET proteins (BRD2, BRD3, BRD4 and BRDT) have been shown to regulate the expression of a variety of genes in the brain. Specifically, BRD2 is essential for neural tube closure and embryonic neurogenesis, but when it is overexpressed, it impairs neuronal differentiation [80, 81].

Epigenetic modification of DNA in the CNS is of extensive concern in 5-methylcytosine (5mC) mediated by DNA methyltransferases and 5-hydroxymethylcytosine (5hmC) converted from 5mC by DNMTs [82–84]. Knockdown of DNMTs, a TET family of dioxygenase proteins, and DNA methylation readers, such as MBD1 and MeCP2, affects the proliferation and differentiation of NPCs by regulating several neurogenic genes to assist the establishment of multiple cell fates in the CNS [85]. During the formation of cerebellar circuitry, the Tet genes and 5hmC robustly exist in developing granule neurons. Higher levels of 5hmC mediated by the TET family predominantly mark the exon start sites of many axon guidance genes and ion channel genes to maintain granule neuron dendritic arborization [86].

#### SWI/SNF complexes

BAF complex-driven chromatin remodeling has been found to be important during neurogenesis and neurodevelopment [87, 88]. In mammals, the BAF complex regulates NSC maintenance or neuronal differentiation depending on its subunit composition. For example, BRG1 and BAF155 deletion impairs neural tube closure [89]. During early neurogenesis, BAF170 has been shown to compete with BAF155 to control euchromatin structure and suppress the expression of Pax6 target genes by directly recruiting the repressor element-1-silencing transcription (REST) corepressor to their promoters, thus repressing indirect cortical neurogenesis and controlling cerebral cortical size and thickness [90]. Researchers have also elucidated that loss of BAF155 and BAF170 breaks the balance between the global repressive (H3K27me2/3) and active (H3K9Ac) epigenetic programs in neural development, impairing epigenetic and gene expression programs of cortical and forebrain development [91], thus leading to abnormal development of the cortex, entire forebrain and related structures (including the olfactory bulb) [92].

Later, the function of the BAF complex in adult neurogenesis has been investigated. In adult neural progenitors, BRG1 is found to interact with Pax6, which then activates a cross-regulatory transcriptional effector network to drive neurogenesis and execute neurogenic fate maintenance. BRG1 deletion in adult NSCs results in the conversion of neural progenitors to gliogenesis [93]. BAF170 has been found to be expressed in radial glia-like (RGL) progenitor cells from adult hippocampi and other adult neurogenic cell types [94]. Loss of either BAF170 [95] or Ctip2 [96] in the adult hippocampus leads to the depletion of the RGL precursor pool and impairs neuronal differentiation.

During brain development, neural migration is required for proper cerebral cortical layer formation, the dysregulation of which leads to several developmental disorders [97]. BAF100a (*Ctip1/Bcl11a*) has been found to be essential in regulating the radial migration of developing cortical pyramidal neurons (PNs). Loss of BAF100a causes abnormal migration of most neurons. Mechanistically, it is found that BAF100 adjusts its downstream effector Sema3c directly to control the polarity and radial migration of PNs, thereby ensuring the correct formation of the epithelial layer [98]. On the other hand, the BAF complex may be implicated in this key process in a Wingless/Int (WNT) signaling-dependent manner. Depletion of the BAF complex (loss of both BAF155 and BAF170) in cortical progenitors and postmitotic neurons leads to the loss of neural migration accompanied by cortical mislamination and abnormal migration of the upper cortical layer (II and III), respectively. In the constructed knockout mouse model, the researchers find that the mechanism of this dysregulation might be the alteration in the cortical gene expression program, leading to the abnormal WNT signaling activity, loss of glial fiber guides, cell adhesion, and defective cell polarization [99]. In addition, BAF53b, a subunit of neuron-specific BAF complexes (nBAF), promotes activity-dependent dendritic outgrowth and proper axonal development by regulating nBAF complexes interacting with CREST to target related genes [100]. Other studies of BAF in neural development and plasticity have been reviewed in [88].

#### INO80 complexes

The INO80 complex plays an important role in early embryonic development and regulates the expression of pluripotency genes. YY1AP1 (YY1 Associated Protein 1) is a component of the INO80 CRC, which is responsible for transcriptional regulation, DNA repair and replication [101]. Knocking out INO80 results in significantly reduced blastocyst formation and decreased expression of pluripotency genes, which are necessary for ES cells to express pluripotency factors [102]. Transformation/transcription domain-associated protein (TRRAP), an essential component of the TIP60–p400 complex and cofactor of HAT, is reported to be essential in regulating programs involved in the cell cycle progression of cortical progenitors during neurogenesis. Loss of TRRAP in the developing cortex disrupts the transcription of E2F cell cycle target genes through impairing HAT recruitment and suppressing related transcriptional machinery [103]. This causes retardation of the cell cycle of cortical NPCs with hindered proliferative capacity, which leads to their inappropriate differentiation in a cell-autonomous manner.

#### ISWI complexes

ISWI protein complexes are involved in DNA replication and repair [104], regulation of transcription [105], and higher-order chromatin structure [41, 106]. Different ISWI complexes mediate the positioning of gene regulatory elements (such as promoters, enhancers, and insulators) on nucleosomes to regulate transcription, proliferation and differentiation. ISWI complexes also play an important role in neurodevelopment [42]. SNF2H and SNF2 L proteins, the core ISWI ATPases in mammals, show different expression patterns in mouse embryos, indicating that they have different roles during development [107]. *Snf2h* (also known as *Smarca5*)-null embryos die in the postimplantation stage [108], while *Snf2l* (also known as *Smarca1*)-null mice survive normally but show excessive and prolonged proliferation of cortical progenitors, leading to enlarged brains [109]. The loss of SNF2H in the developing cerebellum has been found to cause cerebellar hypoplasia and axon symptoms, while the loss of SNF2H in Purkinje neurons after mitosis can lead to neurochemical defects and cognitive changes [110]. SNF2H also mediates lens development and hematopoietic stem cell renewal [111, 112]. SNF2L controls cell cycle exit through FoxG1 dose to regulate neural output and cortical differentiation [109].

#### CHD complexes

The role of CHD protein in neurogenesis during brain development has been extensively investigated [113]. For example, CHD1 is required for embryonic stem cell (ESC) pluripotency by maintaining the “open chromatin state”, and downregulation of CHD1 in ESCs leads to preferential differentiation into the neural lineage [114]. CHD2 regulates embryonic neurogenesis by stimulating the expression of REST to maintain the progenitor pool [115]. CHD3 has an essential role in early brain development [116]. CHD4 depletion promotes astrogenesis without affecting neuronal differentiation in the developing neocortex and disrupts neuronal connectivity in mice [66]. CHD5 is a neuron-specific chromatin remodeler expressed in both the CNS and peripheral tissues of neural origin. The chromodomains of CHD5 bind to H3K27me3 and are essential for neurogenesis, and the depletion of CHD5 blocks neuronal differentiation [117]. CHD7, which is selectively expressed in actively dividing NSCs and progenitors in the SVZ and SGZ, promotes adult neurogenesis by stimulating the expression of Sox4 and Sox11 [118]. CHD8 is reported to be involved in neurodevelopment [119]. It regulates the proliferation and differentiation of NPCs in a stage-specific manner, as knockdown of CHD8 leads to reduced proliferation of NPCs, mis-localization of adult cortical neurons and reduced dendritic arborization [120]. Alternatively, in Chd8 + /del5 (5 bp or 14 bp deletions in Chd8 exon 5) mice, the volume of various brain areas is significantly larger than that in wild-type mice, and the thickness of the cingulate cortex is increased, whereas the deep cerebellar nuclei display decreased relative volume [121].

CHD3, CHD4 and CHD5, as CHD factors within the NuRD complex, have also been implicated in mouse cortical development, during which each CHD has a unique expression distribution in the mammalian brain and regulates distinct and nonredundant aspects, that is, CHD3 and CHD5 are mainly expressed in differentiated neurons in deeper cortical layers (IV-VI) at later embryonic stages and contribute to distinct aspects of neural migration and cortical lamination. CHD5 facilitates the establishment of neuronal polarity and early radial neural migration, whereas CHD3 is necessary for late radial neural migration and proper layer specification [113]. In contrast, CHD4 is strongly expressed in NPCs at the early embryonic stage. In NPCs, CHD4 binds to the promoters of Sox2 (maintaining apical progenitors in proliferative state), Pax6 (regulating cell-cycle length of apical progenitors and differentiation of IPCs), and Tbr2 (involved in IPC proliferation and neurogenesis) genes at much higher levels and increases their expression levels. As expected, ablation of CHD4 results in precocious cell cycle exit of NPCs, loss of IPCs and decreased thickness of the upper cortical layer [113].

Due to the relatively simple structure of the cerebellum, it is considered as a key model in cortical development and cerebellar circuits. CHD4 is robustly expressed in cerebellar granule neurons. CHD4 represses a set of negative regulators in a promoter decommissioning manner, thus driving the presynaptic formation of granule neurons and the neurotransmission to Purkinje cells [122]. Alternatively, deletion of CHD4 in granule neurons impairs dendrite pruning and triggers hyperresponsivity to sensorimotor stimuli [123]. Later, CHD4 has been found to not only suppress genomic accessibility by turning off histone modifications to decommission promoters [122], but also regulate genome architectural by inhibiting the occupancy of architectural protein complex cohesin at enhancer sites [124]. However, in granule cell precursors, CHD7 significantly promotes chromatin accessibility at enhancers of genes involved in granule neuron differentiation, evidenced by increased active histone modification (H3K27ac) levels and RNA polymerase II (RNAPII) binding, thus contributing to terminal neural differentiation [125, 126].

#### Chromatin remodeling in the peripheral nervous system (PNS) neurodevelopment

The PNS is the connection between the CNS and the rest of the body, which consists of all the nerves branching out of the brain and spinal cord. The peripheral nerve is primarily comprised of a bundle of sensory, motor, and autonomic fibers (axons) and Schwann cells (SCs), the supporting glial cells that play a pivotal role in peripheral nerve regeneration. In contrast to the CNS, damaged adult neurons in the PNS retain robust regenerative capacity, which contributes to axon regeneration and sensory-motor functional recovery [127]. The regenerative process requires the induction of widespread chromatin remodeling and a further reactive intrinsic growth transcriptional program [128, 129]. Axon injury in peripheral sensory neurons stimulates HDAC5 nuclear export and further transmission from soma to axon. HDAC5 nuclear export permits the activation of pro-regenerative genes, and its transmission and accumulation at axon tips regulate growth cone dynamics by deacetylating tubulin. These functions together promote axon growth and regeneration [130]. In addition, peripheral axon injury has been reported to induce the expression of TET3 and 5hmC. Notably, TET3 is essential for axon regeneration and behavior recovery by the demethylation of CpG sites of enhancers of regenerative-related genes [131].

**Fig. 3:**
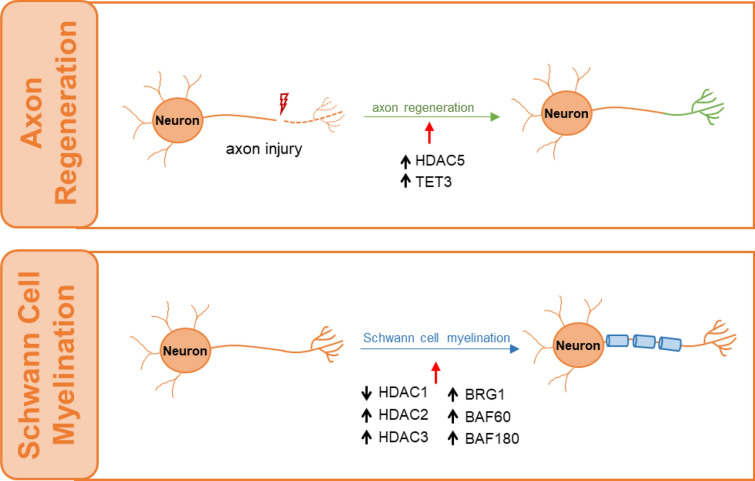
Roles of chromatin remodeling factors in the PNS neurodevelopment. In contract to CNS, damaged adult neurons in PNS retain the axon regeneration, which is facilitated by HDAC5 and DNA dioxygenases TET3. Rapid saltatory conductance is another important physical process in PNS that are carried out by Schwan cells myelination. Chromatin remodeling factors, such as HDAC1, HDAC2, HDAC3, BRG1, BAF60, BAF180, are involved in this process

Myelin sheaths, by supporting axonal integrity and allowing rapid saltatory impulse conduction, are of fundamental importance for neuronal function. SCs produce myelin sheaths and thereby permit rapid saltatory conductance in the vertebrate PNS. Of note, oligodendrocytes form myelin sheaths around the CNS axon in a process that happens postnatally and a single oligodendrocyte can extend its processes to over 100 different axons in the CNS, whereas each SC make up a single myelin sheath on a single peripheral axon in the PNS [132, 133]. Several epigenetic modifications and chromatin remodeling complex are essential for the differentiation and myelination of SCs (Fig. 3). HDAC, CHD, BAF complexes and other chromatin remodeling complexes are required for SC to differentiate and form myelin [134, 135]. For example, HDAC1 and HDAC2 promote SC survival and stimulate the transcription of the myelination program [136], whereas ablation or mutation of HDAC3 induces Krox20 expression and further triggers hypermyelination [137]. In addition, active BRG1 is expressed in SCs during early stages of peripheral myelination. During myelination, the axonal signal neuregulin 1 type III stimulates BRG1 activation through the formation of a complex with the transcription factor NF-κB. Loss of BRG1 inhibits the differentiation of SCs and completely prevents myelin formation [138]. In contrast, polybromo-1 (PBRM1, also known as BAF180), a subunit of PBAF complexes, is present throughout SC development, from the immature to the myelinating stage. Depletion of PBRM1, in contrast to the ablation of BRG1, still retains the normal development of SCs. This may be due to the absence of BAF60 in the PBAF complex [139].

### Chromatin remodeling in learning and memory

Learning and memory, as cognitive functions that help organisms behave adaptively in complex and diverse environments, are of great concern in neuroscience. From different perspectives, memory can be divided into many types, such as declarative and nondeclarative memory, which depend on different brain areas, procedural memory, short-/long-term memory, prospective memory, working memory, etc. [140]. These diverse memory formation and storage processes require the precise transcriptional regulation of memory-related genes and de novo mRNA and protein synthesis. Therefore, an increasing number of studies have shown that chromatin remodeling complexes play important roles in learning and memory processes by dynamically regulating the chromatin state.

#### Histone and DNA modification

Histone methylation is involved in long-term memory formation and consolidation in the hippocampus [141]. During fear learning, H3K4 trimethylation, a transcription active mark, is increased, while H3K9 dimethylation, a silencing mark, is decreased at specific gene promoters, such as the key genes of memory consolidation Zif268 and Bdnf. Mice lacking the H3K4-specific methyltransferase MLL1 show defects in long-term situational fear regulation, prefrontal synaptic plasticity and working memory [142, 143]. In addition, *Utx* deletion causes impaired hippocampal long-term synaptic plasticity (LTP) and synaptic transmission as well as abnormal neuronal morphology, which in turn affects spatial learning and memory [144].

Histone acetylation has been reported to be involved in the early step of long-term memory formation and synaptic plasticity induction. HDAC2 negatively regulates memory formation. Overexpression of HDAC2 in neurons leads to a reduction in synaptic plasticity and memory formation, while HDAC2 deletion causes increased synapse connection and memory facilitation [145]. In contrast, HDAC3, which is highly expressed in the substantia nigra, can enhance LTP in acute hippocampal slices and memory consolidation under contextual fear conditions [146]. Moreover, CBP is implicated in memory formation and consolidation by maintaining the expression of the environmentally driven neuronal activity-related gene *c-fos* [147].

DNMTs not only play essential roles in gene imprinting and transcriptional regulation in the early developmental stages of the CNS but are also critical in adult learning, memory, and cognition [148]. During memory formation, the dynamic regulation of DNA methylation is essential for hippocampal synaptic plasticity. Specifically, the memory suppressor gene protein phosphatase 1 (PP1) is silenced by rapid DNA methylation, while the methylation of *reelin*, a synaptic plasticity gene, is decreased to promote transcriptional activation [149]. Blocking DNMT activity fails to preserve remote memory [150]. Moreover, deletion results in impaired memory extinction and an abnormal increase in hippocampal long-term depression [151].

#### CRCs

In contrast to histones and DNA modification, the mechanism of CRCs involved in learning and memory remains poorly understood. However, several studies have revealed their important roles in regulating memory formation-associated gene expression programs. Specifically, BAF complex-mediated chromatin remodeling in the hippocampus is essential for cognitive functions, as evidenced by the essential roles of the BAF complex in memory formation and learning. As stated above, BAF170 mutant mice show significant damage in adaptive behavior, and loss of BAF170 [94] and CTIP2 [96, 152] causes defects in learning and memory. In another study, BAF53b, a postmitotic neuron-specific subunit of BAF, is found to be required for both hippocampus- [153] and amygdala-dependent [154] memory formation [155]. CHD1 mutation causes severe defects in short- and long-term spatial memory but not in work memory [156]. Conditional knockout of CHD4 in cerebellar granule neurons results in impairment of cerebellar-dependent learning [123]. Moreover, CHD8 knockdown has been found to promote apoptosis and autophagy, resulting in learning and memory impairments [157].

Oligodendrocyte and myelin development are crucial for network integration and are associated with higher brain functions. Accumulating evidences have demonstrated that myelin plasticity and remodeling is necessary for multiple types of memory formation and maintenance and myelin sheaths dysfunctions are associated with cognitive impairments in neurodevelopmental diseases [133]. Therefore, increasing studies have been focusing on the essential role of oligodendrocyte and myelin in learning and memory. For instance, Hasan et al. are the first to reveal that schema-like learning can foster the growth and regeneration of brain myelin, thereby enhancing the learning and memory capacity [158]. Very recently, Bacmeister et al. have shown that motor learning induces phase-specific changes in myelination on behaviorally activated axons that correlate with motor performance, suggesting myelin remodeling is involved in learning [159]. The increased pre-existing myelin sheaths plasticity would influence neuronal activity and learning, and new oligodendrogenesis is also required for several types of learning and memory [160, 161]. Myelin sheaths plasticity is driven by individual neurons but not by oligodendrocytes, and is significantly elevated by Fos promoter activation in behavioral neurons. Although the exact mechanism is unknown, CRCs certainly play an important role in this process. For instance, CHD7, together with the coregulator SOX10, selectively activates myelinogenic factors, such as the novel target genes bone formation regulators Osterix/Sp7 and Creb3l2, thus promoting the terminal differentiation of oligodendrocyte precursor cells (OPCs) to oligodendrocytes (OLs), OL maturation and proper onset of CNS myelination and remyelination [162]. Instead, Olig2 recruits the SWI/SNF chromatin-remodeling enzyme Smarca4/Brg1 to co-occupancy in the cis-regulatory elements of myelination-related genes to initiate OPC differentiation [163]. Moreover, CHD8 is mainly critical for OPC development, proliferation, and survival by recruiting the H3K4 methyltransferase MLL/KMT2 to activate the transcriptional program [164].

## Dysfunction of chromatin remodeling in neurodegenerative diseases

Imbalance of neuronal chromatin structure and the ensuing alterations in gene expression are detrimental to brain function. Given the critical role of chromatin remodeling in brain development and function, such as learning and memory, it may contribute to memory-related brain disorders. Many enzymes responsible for distinct histone modifications, DNA methylations and chromatin remodeling have been associated with neurodegeneration. For example, HDAC2 and HDAC3 are two of the most highly expressed HDACs in the substantia nigra, making them potential therapeutic targets for neurodegenerative disorders. Knockout of LSD1 in the adult brain causes neuronal death in hippocampal and cortical neurons, triggering neurodegeneration [165]. Discussed below are recent evidence implicating the dysfunctions of chromatin remodeling in the pathological process of neurodegenerative diseases, including Alzheimer’s disease (AD), Parkinson's disease (PD), Huntington’s disease (HD) and Amyotrophic lateral sclerosis (ALS), with a strong focus on histone modifications (Table 2).

**Table 2:** The dynamic changes of chromatin remodeling modification/factors in neurodegenerative diseases

Neurodegenerative diseases	Chromatin remodeling modification/factors	Changes in Diseases	References
Alzheimer’s disease (AD)	H4 acetylation	Decreased	[166–168]
	H3K27ac	Global increased	[169]
	H3K9me2	Increased	[177]
	DNA methylation	Not certain	[207, 210, 211]
	HDAC1	Decreased	[174]
	HDAC2	Increased	[230]
	HDAC3	Increased	[172, 173]
	HDAC6	Increased	[171]
	EHMT1	Increased	[177]
Parkinson’s disease (PD)	H3K14ac	Increased	[182]
	H3K18ac	Increased	[182]
	HDAC2	Increased	[231]
Huntington’s disease (HD)	H3K9me3	Increased	[188]
	H3K4me3	Decreased	[189]
	HDAC1	Increased	[193]
	HDAC2	Increased	[232]
	HDAC3	Increased	[192, 193]
	HDAC4	Increased	[194]
	H2AFY	Increased	[195]
Amyotrophic lateral sclerosis (ALS)	H3K14ac	Decreased	[196]
	H3K56ac	Decreased	[196]
	H4K12ac	Increased	[196]
	H4K16ac	Increased	[196]
	HDAC11	Decreased	[197]
	HDAC2	Increased	[197]
	H2B Thr129 and Ser10 phosphorylation	Decreased	[196]
	H3 Thr129 and Ser10 phosphorylation	Decreased	[196, 200]
Amyotrophic lateral sclerosis (ALS)	H3K9me3	Increased	[201]
	H3K27me3	Increased	[201]
	H4K20me3	Increased	[201]
	H2A K119 ubiquitination	Decreased	[202]
	DNMT1	Increased	[214]
	DNMT3a	Increased	[214]
	Mitochondrial DNA methylation	Increased	[216]
	CHD1	Decreased	[221]
	CHD2	Decreased	[221]
	nBAF	Decreased	[224]

### Histone modifications in AD

The role of HDACs and the change in histone acetylation in AD have been an intense field of research. Dramatically decreased level of brain H4 acetylation has been observed in different mouse models of AD, which can be restored by HDAC inhibitors [166–168]. In addition, an overlap between H3K27ac levels and AD GWAS regions has been identified in a histone acetylome-wide association study [169]. These data provide evidence for the link between histone modifications and AD, although whether it is a causal factor or a result of AD is unclear. Identifying the role of each HDAC in AD is vital to the development of specific HDAC inhibitors for AD treatment. Several research groups are exploring the potential of HDAC inhibitors as AD therapeutics. In AD models, class I HDACs seem to be essential for the observed effects of HDAC inhibitors. There is evidence that levels of HDAC2 [170] and HDAC6 [171] rise in postmortem brain tissue from AD patients compared with controls, while HDAC3 has been found to regulate the expression of proteins associated with AD pathophysiology [172] and memory formation [173]. The role of HDAC1 in AD has only been studied recently. Tsai et al. found that in AD mice, HDAC1 levels decrease and HDAC1 activity is impaired, which exacerbates 8-oxoguanine (8-oxoG) DNA lesions. Pharmacological activation of HDAC1 could stimulate 8-oxoGDNA glycosylase 1 (OGG1) activity, reduce 8-oxoG lesions and improve cognition in AD mice, thus highlighting the therapeutic potential of HDAC1 activation in brain neurodegeneration [174]. It should be noted that this study measures not only HDAC levels but also their activation state. In contrast, very recently, the first HDAC positron emission tomography (PET) data from AD patients using [^11^C] martinostat, a PET tracer that recognizes class I HDACs in the brain, revealed global reduced HDAC I levels in AD-affected brain regions where amyloid and tau are high [175]. However, these contradictory findings can be explained, as the martinostat tracer recognizes HDACs in the brains of living people and does not distinguish between particular class I isoforms. Furthermore, most previous research on HDAC has been performed in mouse models. Even by using postmortem samples, different brain regions may be examined. Although the finding of low levels of HDAC I in AD questions HDAC inhibition in AD trials, it highlights HDAC I reduction as an element associated with AD pathology and emphasizes the importance of developing isoform-specific HDAC inhibitors in curing AD.

Changes in histone methylation have been reported in postmortem brain tissues from human donors with AD [176], suggesting that targeting histone methylation would be a new avenue in AD treatment. H3K9me2 is one of the most extensively studied histone methylation marks associated with AD. Levels of H3K9me2 in the prefrontal cortex and hippocampus of aged FAD mice as well as in postmortem tissues from AD patients are significantly elevated [177]. Increased levels of the histone methyltransferase EHMT1 but not EHMT2 are observed in the postmortem prefrontal cortex of AD patients.

### Histone modifications in PD

Parkinson's disease (PD) is the second most common age-related neurodegenerative disease and is characterized by involuntary resting tremor, stooped posture, weakness of the limbs and some nonmotor symptoms, such as cognitive impairment and sleep disorders. It is associated with degeneration of dopaminergic neurons due to intracytoplasmic inclusions composed of aggregated α-synuclein, ubiquitin-protein and damaged nerve cells. Most PD cases are sporadic and do not have a clear genetic link. Increasing evidence supports that epigenetic changes are involved in PD progression. However, current studies show large inconsistencies in histone acetylation and HAT/HDAC changes in PD patients. Histone acetylation is disease-dependently altered in PD [178]. α-Synuclein has been shown to have increased expression in PD patients, the accumulation of which will cause a misbalance in the actions of HATs/HDACs, thus inhibiting histone acetylation and deregulating gene transcription [179, 180]. Although both HDACs and histones are suggested to be more involved in aggregate formation than in gene expression regulation in PD [181], a recent study shows that enhanced gene transcription in the postmortem PD primary motor cortex is mainly due to increased H3K14 and H3K18 acetylation [182]. In another study, dopaminergic neurons from PD patients show much higher histone acetylation than control neurons, which is mainly caused by the downregulated HDACs but not by the unchanged KATs [183].

### Histone modifications in HD

Huntington’s disease (HD) is an autosomal dominant neurodegenerative disorder caused by a polyglutamine repeat expansion within the huntingtin protein, and transcriptional dysregulation has been proposed to be an early event in HD pathogenesis. Recent studies have demonstrated that mutant huntingtin alters HDAC activity, the dysfunction of which might be an underlying mechanism of transcriptional dysregulation in HD. In addition, other epigenetic modifications, such as histone methylation, ubiquitination, phosphorylation, DNA modifications, and the expression of histone variants, have all been implicated in HD [184–187]. For instance, increased H3K9me3 in heterochromatin domains and decreased H3K4me3 levels at the promoters of downregulated genes have been observed in HD mouse models and HD postmortem brain tissues, and reducing H3K9me3 levels slows disease progression, while increasing H3K4me3 levels is protective in HD mouse models [188, 189]. Histones are hypoacetylated in different HD mouse models as well as cell lines. In addition, H3, H4, CBP and many other histone modifying proteins have been shown to be sequestered in Htt aggregates in transgenic mice and in the brains of HD patients [190]. The inhibition of HDACs, such as HDAC1 and HDAC3, has been shown to rescue cognitive deficits, motor deficits or metabolic dysfunctions in different mouse models of HD, thus offering a potential therapeutic strategy for future study [191–193]. Knocking down HDAC4 has been shown to improve behavioral and neuropathological phenotypes in HD mice [194]. For histone variants, H2AFY, which encodes the histone variant macroH2A1, is found to be increased in the cellular blood of HD patients [195]. These studies suggest that changes in histone methylation and acetylation marks occur in HD, and deeper insight into these changes could help the development of future therapies for this disease.

### Histone modifications in ALS

ALS is the third most common neurodegenerative disorder and is characterized by the selective loss of both upper and lower motor neurons. Approximately 10% of all ALS patients suffer from familial ALS that is associated with a number of genetic variants, including superoxide dismutase 1 (SOD1), fused in sarcoma (FUS), TAR DNA binding protein 43 (TDP-43), and chromosome 9 open reading frame 72 (C9orf72). Recent evidence shows a link between ALS and epigenetics. The global changes in histone acetylation have been characterized in yeast ALS proteinopathy models. Yeast overexpressing human FUS results in hypoacetylation of H3K14 and H3K56, while yeast overexpressing TDP-43 reveals hyperacetylation of H4K12 and H4K16, suggesting that each proteinopathy shows distinct changes in histone modification profiles [196]. In addition, the role of HDACs in ALS has also been studied. Decreased HDAC11 mRNA and increased HDAC2 mRNA levels have been reported in postmortem ALS brain and spinal cord tissues [197]. Other evidence for the association of HDACs with ALS comes from different mouse models. For example, HDAC1 aggregates into nuclear foci in a FUS knock-in mouse model, and this abnormal HDAC1 subcellular distribution might lead to toxicity in motor neurons [198]. In another study, the phosphorylation of HDAC1 results in accumulation in the nucleus, which is neuroprotective in an ALS mouse model [199]. Histone phosphorylation, methylation, ubiquitylation and many enzymes responsible for these modifications have also been implicated in ALS. For example, FUS knockdown increases H3 phosphorylation in different cellular models [200], while FUS overexpression in yeast reduces H2B and H3 phosphorylation on Thr129 and Ser10, respectively [196]. The dipeptide repeat expansions in C9orf72 in ALS patients increases H3K9me3, H3K27me3 and H4K20me3 levels in the brain [201] but impairs H2A ubiquitination at K119 in the spinal cord [202].

### DNA methylation in neurodegenerative diseases

DNA methylation, the best-known epigenetic mark, has attracted much attention in the pathogenesis of age-related neurological disorders [203, 204]. Dysregulation of DNA methylation in neurodegenerative diseases, such as AD and PD, has been well documented [205, 206], which helps us understand the basis of these diseases. However, by assessing global DNA methylation as a percentage of 5-methylcytosine (5mC)/5-hydroxymethylation using brain samples, previous studies report conflicting findings regarding the association between global DNA methylation and AD, with some authors describing lower methylation levels in AD cases [207–209] and others reporting no alteration [210] or increase [211] in methylation in AD subjects compared to the control. Altered DNA methylation patterns, misexpression of disease-associated genes and mutations in DNMTs and methyl-CpG binding proteins have been observed in PD [212], AD, HD and ALS [213]. For instance, the levels of Dnmt1, Dnmt3a and 5-methylcytosine are increased in the brain and spinal cords of ALS patients, and unsurprisingly, global changes in DNA methylation and hydroxymethylation have been observed in postmortem spinal tissue of ALS patients [214, 215]. Mitochondrial DNA methylation is also increased in SOD1 mice [216]. Overall, DNA methylation appears to play an essential role in the pathology of many neurodegenerative diseases.

### CRCs in neurodegenerative diseases

Aside from histone modifications and enzymes targeting histone modifications, some ATP-dependent CRCs have also been associated with neurodegenerative disease. It should be noted that, given the essential roles of CRCs in neurogenesis and neurodevelopment, it is not surprising that abnormal function of subunits of these CRCs will elicit neurodevelopmental diseases. For instance, studies have revealed that heterozygous mutations in distinct subunits of the BAF complex will lead to many neurodevelopmental disorders that show similar intellectual impairment [217]. Pathogenic variants in CHD proteins contribute to the development of a range of neurological disorders, such as CHD1 in Pilarowski-Bjornsson syndrome [218], CHD3 in Snijders Blok-Campeau syndrome (SNIBCPS) [116], CHD4 in Sifram-Hitz-Weiss syndrome [219], CHD7 [220] and CHD8 [119] in Autism Spectrum Disorder (ASD). Compared to neurodevelopmental disease, relatively few studies have examined the association between CRCs and neurodegenerative diseases. However, mounting evidence shows that changes in the nuclear localization or recruitment of CRCs are important for the maintenance of neuronal integrity. Loss of CHD1 enhances TDP-43-mediated neurodegeneration in a Drosophila model of ALS, while CHD2, which physically interacts with TDP-43, is significantly downregulated in the temporal cortex of ALS patients [221]. These findings indicate that by interfering with CHD1/CHD2, TDP-43 impairs chromatin dynamics at stress genes, thus reducing the protective stress response and promoting neurodegeneration. In addition, FUS interacts with nBAF proteins, and mutations in BAF subunits are also related to ALS [222]. Mutant TDP-43 or FUS has been found to reduce Brg1 levels in cultured motor neurons [223, 224]. Importantly, loss of nBAF subunits occurs in spinal motor neurons from both familial and sporadic ALS autopsy specimens, suggesting that dysregulation of nBAF chromatin remodeling is an important mechanism of neuronal dysfunction in multiple forms of ALS [224]. Overall, these studies indicate that CRCs, which do not specifically affect histone modifications, also contribute to pathologies of ALS and other neurodegenerative diseases, thus highlighting the critical need for the inclusion of chromatin remodeling in neurodegenerative disease research.

## Chromatin remodeling regulation by small molecules for the treatment of neurodegenerative diseases

As mentioned above, proper chromatin regulation is essential for gene expression in the brain, and imbalance of neuronal chromatin structure and the ensuring changes in gene expression are detrimental to brain functions. Chromatin remodeling can be accomplished by covalent modification of histones or by the action of ATP-dependent remodeling complexes. Some histone modifications directly contribute to chromatin structure, and enzymes, including those directly affecting histone modifications and ATP-dependent CRCs, can also function in the regulation of chromatin structure. Notably, other factors may also be included, such as PARP1-mediated chromatin remodeling. Since derangement of these various epigenetic mechanisms of chromatin remodeling has been implicated in several neurodegenerative diseases, drugs targeting all these underlying epigenetic defects might be useful as therapeutic agents in various neurodegenerative diseases.

In the postgenomics epigenetic era, because epigenetic mechanisms are pharmaceutically accessible and largely reversible, inhibitors of chromatin remodeling factors, such as epigenetic drugs, have made great progress. Currently, 14 types of epigenetic drugs have been approved. Most small molecule inhibitors are in the clinical stage for cancer treatment, and some inhibitors that are used for the treatment of neurodegenerative diseases are in the early clinical stage. Based on the targeted substrates, these small molecule modulators may be divided into different groups. In this section, we will give an overview of the recent progress that has been made in this field (Table 3).

**Table 3:** The substrate specificity, related neurological diseases and clinical trials of small molecular inhibitors

HDACi				
Class	Compound	HDAC specificity	Related neurological diseases and clinical trials	References
Hydroxamate	Vorinostat (SAHA)	Class I, II	Alzheimer’s disease (Phase I, NCT03056495), Parkinson’s disease, Huntington’s disease, Spinal muscular atrophy, Frontotemporal dementia	[179, 237–241]
Hydroxamate	Panobinostat (LBH589)	Class I, II, IV	Huntington’s disease, Spinal muscular atrophy	[243–245]
Hydroxamate	Trichostatin A (TSA)	Class I, IIb	Amyotrophic lateral sclerosis, Spinal muscular atrophy, Parkinson’s disease	[246–249]
Fatty acid	Sodium butyrate (NaBu)	Class I, II	Alzheimer’s disease, Huntington’s disease, Parkinson’s disease, Spinal muscular atrophy, Polyglutamine diseases (e.g., SCA3, SBMA)	[179, 185, 252–257, 261]
Fatty acid	Valproic acid (VPA)	Class I, II	Alzheimer’s disease (Phase I, NCT01729598, Phase II, NCT00088387, Phase III, NCT00071721), Huntington’s disease (Phase II, NCT00095355), Parkinson’s disease, Amyotrophic lateral sclerosis (NCT00136110; Phase II, NCT03204500)	[262–267]
Fatty acid	Sodium Phenylbutyrate (4-PBA)	Class I, II	Alzheimer’s disease (Phase II, NCT03533257), Huntington’s disease (Phase II, NCT00212316), Parkinson’s disease (Phase I, NCT02046434),	[167, 268, 270, 271]
Fatty acid	AMX0035	Class I, II	Amyotrophic lateral sclerosis (Phase II, NCT03127514, Phase III, NCT05021536); Alzheimer’s disease (Phase III, NCT03533257)	[272]
Benzamide	Entinostat (MS-275)	Class I	Alzheimer’s disease	[273]
Benzamide	RGFP966	HDAC3	Huntington’s disease, Alzheimer’s disease	[172, 274]
Benzamide	RGFP109	HDAC1/3	Huntington’s disease, Parkinson’s disease	[193, 275]
Benzamide	M344	class I and IIb	Spinal muscular atrophy, Alzheimer’s disease	[276, 277]
Benzamide	K560	HDAC1, HDAC2	Parkinson’s disease	[278]
Benzamide	Nicotinamide (NAM)	Class III	Friedreich's ataxia (Phase II, NCT01589809), Parkinson Disease (NCT03568968, Phase II, NCT04044131), Alzheimer’s disease (Phase II, NCT03061474, Phase II, NCT04044131)	[279]
Benzamide	HDACi 4b and 136	HDAC1, HDAC3	Huntington’s disease	[280, 281]
Cyclic tetrapeptide	Romidepsin (FK228)	HDAC1, HDAC2	Alzheimer’s disease	[170, 282]
Miscellaneous	mercaptoacetamide-based compound W2	Class II	Alzheimer’s disease	[283]
Miscellaneous	CM-144	HDACs, DMNT1, G9a and PDE5	Alzheimer’s disease	[284]
Miscellaneous	CKD-510	HDAC6	Charcot-Marie-Tooth disease (Phase I, NCT04746287)	NA
Miscellaneous	CKD-504	HDAC6	Huntington’s disease (Phase I, NCT03713892), Alzheimer’s disease	[285]
Miscellaneous	Resveratrol (RVT)	HDAC, DNMT and LSD1 inhibitor	Alzheimer’s disease, Amyotrophic lateral sclerosis (Phase II, NCT04654689), Spinal muscular atrophy	[288–292]
HAT activator				
Compound		HAT specificity	Related neurological diseases and clinical trials	
CTPB		p300	Parkinson’s disease	[309]
HMTi				
Compound		HMT specificity	Related neurological diseases and clinical trials	
BIX-01294		G9a	Alzheimer’s disease	[177, 302, 304]
UNC0642		G9a/GLP	Alzheimer’s disease	[177, 303, 304]
HDMi				
Compound		HDMT specificity	Related neurological diseases and clinical trials	
Vafidemastat (ORY-2001)		LSD1	Alzheimer’s disease (Phase II, NCT03867253)	[306]
GSK-J4		KDM6A/B, KDM5B/C	Parkinson’s disease	[307]
BRD domain inhibitor				
Compound		BRD specificity	Related neurological diseases and clinical trials	
JQ1		BRD2, BRD3, BRD4, BRDT	Alzheimer’s disease, Parkinson’s disease, levodopa-induced dyskinesia, Amyotrophic lateral sclerosis	[313–316]

### HDAC inhibitors in neurodegenerative diseases: preclinical and clinical study

HDAC inhibitors are small molecules that have been identified from natural sources and developed synthetically. They restrain HDAC activity and maintain chromatin in the decondensed state, which further affects transcription by inducing acetylation of histones, transcription factors and other proteins regulating transcription. These HDAC inhibitors (HDACi) vary in structure, specificity and biological activity and can be divided into four classes based on the chemical structure, which include hydroxamic acid derivatives, short-chain fatty acid derivatives, synthetic benzamides, and cyclic peptides. While the majority of HDACi work broadly on all HDAC isoforms but not the SIRT enzymes (pan inhibitors), certain HDACi may selectively inhibit specific HDACs. To date, 20 different HDACi have been evaluated in clinical trials for the treatment of a broad range of cancers [225, 226]. Among them, vorinostat (SAHA), belinostat (PXD101), panobinostat (LBH589), romidepsin (FK228) and mocetinostat (MGCD0103) have been approved by the Food and Drug Administration (FDA) for the treatment of various cancers, including T-cell lymphoma and multiple myeloma [227], while chidamide (CS055) selectively inhibits the activity of HDAC1, HDAC2, HDAC3 and HDAC10, and is the first oral active HDACi of the benzamide class approved in China for the treatment of relapsed and refractory peripheral T-cell lymphoma (PTCL) in 2014 and breast neoplasms in 2019.

As mentioned above, impairments in HDAC functions and associated pathways as well as transcriptional dysfunctions have been implicated in the pathogenesis of AD, PD, HD, ALS and other neurodegenerative diseases [228, 229]. For example, increased HDAC2 levels have been found in the brains of patients with AD [230], PD [231], HD [232] and ALS [197]. In addition, acetylation balance is dramatically impaired in neurodegenerative diseases [233]. These studies have led to the increasing exploration of HDAC inhibitors in different preclinical models of neurodegeneration regarding their potential therapeutic applications for the treatment of neurodegenerative diseases [228, 229, 234–236], and some successful treatments in animal models have been translated into clinical trials. Many HDACi are currently in clinical trials for treating distinct neurodegenerative diseases.

#### Hydroxamic acid-based HDACi

Hydroxamic acids are the largest class of HDACi, mainly including SAHA, LBH589, PXD101, trichostatin A (TSA), dacinostat (LAQ824), m-carboxycinnamic acid (CBHA), azelaic bis-hydroxamic acid (ABHA), givinostat (ITF2357) and PCI-34051. Notably, three of the six anticancer HDAC inhibitors approved by the FDA are hydroxamic acids, including SAHA, LBH589 and PXD101.

As a class I/II HDAC inhibitor, SAHA has been reported to ameliorate the progressive neurodegeneration involved in different models (e.g., mice, rats, Drosophila and humans) of various neurodegenerative disorders, such as AD [237], PD [179, 238], HD [239], spinal muscular atrophy (SMA) [240], and frontotemporal dementia (FTD) [241]. For example, SAHA has been found to be effective in restoring contextual memory deficits through inhibition of HDAC6 in a mouse model of AD [237]. A combination of SAHA and tadalafil, an inhibitor of phosphodiesterase-5 (PDE-5), shows a synergistic effect on both reducing amyloid pathology and alleviating cognitive deficits in AD mice [242]. When complexed with cyclodextrins, SAHA can cross the blood‒brain barrier and improve motor impairment in a mouse model of HD, possibly acting by ameliorating the transcriptional changes in HD [239]. A study in both cell culture and transgenic *Drosophila* models of PD shows that administration of SAHA restores α-synuclein-dependent neurotoxicity due to an interaction between histones and α-synuclein [179]. Despite the abovementioned promising therapeutic effects of SAHA in different animal models of neurodegenerative disease, it should be noted that certain studies state the limited therapeutic benefits of SAHA owing to its toxicity [239]. In a phase I trial, the safety and tolerability of SAHA to treat AD patients has been evaluated (NCT03056495).

LBH589, as a novel broad-spectrum HADC (class I/II/IV) inhibitor, is a cinnamic hydroxamic acid analog that is approved by the FDA in 2015. As LBH589 is an orally bioavailable HDACi and is at least tenfold more potent than SAHA, its therapeutic potential for neurodegenerative diseases has been assessed. LBH589 has been shown to cross the blood‒brain barrier, induce histone hyperacetylation and ameliorate neuropathological phenotypes in two preclinical mouse models of HD [243]. Two years later, in another study, interventional treatment in early presymptomatic phenotypes of HD with LBH589 has been shown to significantly improve behavioral changes [244]. Recently, LBH589, together with a splice-switch antisense oligonucleotide, has also shown beneficial effects for SMA in preclinical studies, showing induction of H4 acetylation of the SMN2 locus and increased SMN2 splicing and SMN protein expression [245].

TSA, a natural derivative of dienodydroxamic acid, is an antifungal antibiotic that is reported to selectively inhibit class I/IIb HDAC. TSA has also been shown neuroprotective effects in many studies. TSA treatment in APP/PS1 mice inhibits the reduction of H4 acetylation, leading to an increase in memory formation [166]. TSA shows beneficial effects for treating ALS. Yoo et al. found that it decreases the level of motor neuron cell death and ameliorates muscle atrophy and neuromuscular junction denervation in an SOD1 ALS mouse model [246]. Very recently, Bennett et al. report that TSA suppresses FUS-associated toxicity and relieves growth suppression by controlling histone acetylation on specific modification sites using a FUS ALS/FTD yeast model [247], which further supports the clinical potential of TSA in the treatment of ALS. In another study, TSA treatment induces the expression of SMN2 and improves survival and motor pathology in SMA mice [248]. Neuroprotective effects of TSA have also been reported in a mouse model of PD through upregulation of the expression of tyrosine hydroxylase and brain-derived neurotrophic factor [249].

#### Short-chain fatty acid-based HDACi

The second class of HDACis are short-chain fatty acids, which mainly include sodium butyrate (NaBu), valproic acid (VPA), phenylbutyrate (4-PBA), and AR-42. Compared with hydroxamic acid-based HDACis, short-chain fatty acids have good penetration into the brain, making them potential candidates for treating neurodegenerative diseases. VPA, NaBu and 4-PBA have validated therapeutic utility in the treatment of different neurodegenerative disorders, such as AD, PD, HD, and SMA [250].

NaBu, one of the first discovered HDACis, belongs to the short-chain fatty acid class of HDACis. NaBu is a very potent HDACi of class I/II HDAC enzymes. Because NaBu is capable of crossing the blood‒brain barrier and increasing the level of H3 acetylation in the brain, its potential use in the intervention of neurodegenerative diseases [251] is of longstanding interest. However, due to its poor pharmacokinetic properties, butyrate is not clinically viable as a drug. NaBu is reported to restore learning and related memory in Ck-p25 mice with AD pathology and improve social behavior in an AD mouse model [252, 253]. Treatment with NaBu in different HD mouse models shows improvement in motor impairment, attenuated neurodegenerative phenotypes and enhanced survival [254, 255]. Administration of NaBu decreases neuronal death in response to α-synuclein in cell culture and transgenic flies of PD [179]. NaBu can ameliorate neuromuscular phenotypes of SMA [256, 257]. Moreover, NaBu has also been shown to have possible therapeutic effects in different animal models of polyglutamine (polyQ) diseases [185]. For instance, spinocerebellar ataxia type 3 is the most common form of spinocerebellar ataxia worldwide and is characterized by progressive ataxia, spasticity and ocular movement abnormalities [185, 258–260]. Chou et al. found that NaBu ameliorates ataxic symptoms in a transgenic mouse model of SCA3 [261]. Spinal and bulbar muscular atrophy (SBMA) is an inherited motor neuron disease caused by the expansion of a polyQ tract within the androgen receptor, and oral administration of NaBu has been shown to improve motor impairment in SBMA mouse models through the restoration of histone acetylation [257].

VPA is a short-chain fatty acid that inhibits both class I and II HDACs. The therapeutic ability of VPA has been demonstrated in different animal models of AD, PD and ALS [262]. Early daily injection of relatively low doses of VPA in a transgenic AD mouse model significantly reduces Aβ plaque deposition and improves memory deficits, which could be attributed to the inhibition of GSK-3β-mediated γ-secretase cleavage of APP [263]. A latter study using a different AD mouse model shows that VPA treatment at early and late-symptomatic stages of AD decreases escape latencies [264]. When injected into a transgenic mouse model of ALS, VPA prevents histone deacetylation in the spinal cord, shows motoneuronal protection, retarded muscular atrophy to some extent and delays but does not prevent neuromuscular denervation [262]. Several clinical trials have been performed to analyze the effectiveness of VPA in the management of various neurodegenerative diseases. For example, VPA has been reported to change clusterin expression in elderly people with AD in a phase I study (NCT01729598). However, a phase III clinical trial (NCT00071721) reports the failure to delay agitation, psychosis, slow cognitive or functional decline in moderate AD patients and significant toxic effects [265]. Divaproex, a stable coordination compound comprised of sodium valproate and VPA, has been in a phase II clinical trial to evaluate its neuroprotective effects in patients with AD (NCT00088387) and HD (NCT00095355). In addition, a clinical trial of VPA in ALS patients (NCT00136110) shows that when given alone, VPA fails to show a beneficial effect on survival or disease progression [266]. Later, a phase II clinical trial (NCT03204500) shows that when VPA is given together with lithium, increased survival and neuroprotection effects are observed in ALS patients; however, the trial stops after 21 months due to late adverse events [267].

Another short-chain fatty acid drug, 4-PBA, is a derivative of the aromatic fatty acid butyrate. In the Tg2576 AD mouse model, daily injection of 4-PBA is reported to reverse spatial memory impairment by normalizing tau hyperphosphorylation in the hippocampus, with restored hippocampal H4 acetylation and dendritic spine density defects [167]. In a study with a mouse model of PD, 4-PBA treatment significantly reduces the diminution of dopamine and loss of tyrosine hydroxylase-positive neurons in the substantia nigra [268]. Cotreatment of 4-PBA with the catalytic antioxidant AEOL 10,150 in ALS mice exerts additive neuroprotective effects, as evidenced by improved motor functions, enhanced survival, and reduced oxidative damage in the lumbar spinal cord [269]. To date, 4-PBA has been assessed in phase I and II clinical trials in AD, HD and PD. A dose-finding study of 4-PBA has been performed in HD patients showing that low doses of PBA are sufficient to correct transcriptional abnormalities seen in the blood of HD patients [270] and increase renal excretion of potentially neurotoxic indole metabolites, as seen in the recent phase II study with patients with early symptomatic HD [271]. The safety, tolerability and clinical impact of 15 g daily of 4-PBA has been tested in HD patients in a phase II trial (NCT00212316). 4-PBA is currently in a phase I clinical trial assessing its ability to remove α-synuclein from the brain into the bloodstream (NCT02046434). In addition, AMX0035, a fixed combination of 4-PBA and taurousodeoxycholic acid, is designed to reduce neuronal death and degeneration. A phase II clinical trial for AMX0035 in patients with ALS has been completed recently (NCT03127514) [272]. It is currently in a phase II and III trial to evaluate its safety and efficacy for the treatment of AD (NCT03533257) and ALS (NCT05021536), respectively.

#### Benzamide-based HDACi

Apart from hydroxamic acids and short-chain fatty acids, benzamide-containing compounds represent another class of HDACi with clinical potential. Entinostat (MS-275) is a synthetic benzamide derivative that blocks the activity of HDAC Class I (isoform 1/2/3). Of note, oral administration of entinostat reduces amyloid plaque deposition in the hippocampus and cortical areas of a transgenic mouse model of AD [273]. RGFP966, a benzamide HDAC3 inhibitor, has been shown to improve motor deficits in the rotarod and in the open field in a transgenic HD mouse model, mainly through the induction of macrophage migration inhibitory factors, which leads to the activation of glial cells [274]. RGFP966 treatment of 3xTg AD mice also shows beneficial effects, which leads to reversed AD-related pathologies and improved cognition of AD mice [172]. RGFP109, a brain-penetrant inhibitor of HDAC1/3, has been shown to offer benefits for attenuating motor deficits in HD [193] as well as in PD [275]. SMN2 represents a promising target for SMA therapy. Treatment of fibroblast cells derived from an SMA patient with M344, a benzamide HDACi of class I and IIb, results in significant upregulation of SMA2 protein expression [276], indicating that M344 may be a promising candidate for a causal therapy of SMA. Importantly, M344 affects many AD-related key genes that are involved in early- and late-onset AD pathogenesis and attenuates cognitive decline in an AD mouse model [277]. Another example of a benzamide class HDACi is K560 [2-amino-5(tiophen-2yl) benzamide]. It exhibits a stronger inhibition toward HDAC1 and HDAC2. K560 has been shown to attenuate cell death through the sustained expression of an antiapoptotic protein, X-linked inhibitor of apoptosis, in differentiated SH-SY5Y cells, and K560 treatment leads to dopaminergic neuroprotection in a mouse model of PD [278].

Nicotinamide (NAM), an inhibitor of class III HDACs that readily crosses the blood‒brain barrier, is found to be effective in preclinical Friedreich's ataxia (FA) models and has been tested in clinical trials in FA patients for dose escalation (NCT01589809). NAM is described as a protective agent in PD mainly through the activation of SIRT1 [279]. Recently, patients with newly diagnosed PD are recruiting for a clinical trial to study whether NAM supplementation will correct NAD deficiency and thereby slow the progression of PD symptoms (NCT03568968). A recent phase II trial with a combination of N-acetylcysteine, L-carnitine tartrate, nicotinamide riboside and serine recruits patients to study metabolic improvements in AD and PD subjects (NCT04044131), and another phase II trial using NAM for AD treatment is also ongoing (NCT03061474). In addition, recently developed benzamide-derived compounds, such as HDACi 4b and 136, which tend to target HDAC1 and HDAC3, have been shown to be effective in reversing the expression of HD-related genes [280, 281].

#### Cyclic peptide-based HDACi

The cyclic peptide-based class represents the most potent and most structurally complex group of HDACi, comprising both epoxyketone- and nonepoxyketone-containing tetrapeptides. Cyclic peptide HDACis mainly include romidepsin (FK228), apicidin, trapoxin, depudesin, CHAP, spiruchostatin A and largazole. FK228, a bicyclic class I selective HDACi, is the first FDA-approved peptide-based drug for the treatment of cutaneous T-cell lymphoma (CTCL) and PTCL. As an HDAC1 and HDAC2 inhibitor, FK228 has been shown to play an essential role in cognitive loss in AD [170, 282].

#### Other HDACi

Other HDACis are miscellaneous based on chemical structure. A novel class II HDACi, mercaptoacetamide-based compound W2, has been found to reduce Aβ levels and improve cognition in 3 xTg AD mice [283]. CM-144 acts as a combined inhibitor of HDACs, DMNT1, G9a and PDE5. Chromic treatment of Tg2576 AD mice with CM-144 diminishes AD-related pathologies, rescues hippocampal synaptic impairment and reverses the cognitive deficits of AD mice [284].

CKD family members have been exploited recently by Chong Kun Dang Pharmaceutical Corp in Korea. Among them, CKD-510 is currently in a phase I trial to treat Charcot-Marie-Tooth disease (NCT04746287), an inherited neurological disease characterized by a slowly progressive degeneration of the muscles in the feet, legs and hands. CKD-504, a specific inhibitor of HDAC6, is currently in a phase I trial to treat HD (NCT03713892). Moreover, CKD-504 can reduce pathological tau protein and rescue memory impairment of AD mice by effectively regulating global acetylation in brain [285].

Resveratrol (RVT), a phytoalexin produced naturally by several plants, modulates several epigenetic targets, including HDAC, DNMT and LSD1 [286]. RVT-mediated neuroprotection has been observed in several studies on AD [287, 288]. For example, Chen and colleagues demonstrate a protective effect of RVT in mixed cortical cultures against Aβ toxicity, as it inhibits microglial NF-κB signaling by activating SIRT1 [289]. Multiple studies have also shown the beneficial effects of RVT in ALS [290] by activating SIRT1. In another study, Dayangac-Erden and coworkers show that RVT treatment increases SMN2 mRNA and protein levels in 3813 cell lines, thus showing therapeutic potential to treat SMA [291]. A phase II trial tests the impact of the combined treatment of liposomed polyphenols resveratrol and curcumin with G04CB02 on ALS patients (NCT04654689).

The mechanism of action of HDACi in the treatment of neurodegenerative disorders is currently unclear. Notably, there have been studies that show contradictory effects of HDACi. For example, Wang et al. find that TSA increases dopaminergic cell death caused by MPP + or rotenone treatment [292]. In another study, VPA is also reported to be an apoptosis inducer in hippocampal neurons [293]. Therefore, the effects of HDACi in different neurodegeneration models are complex. Plausibly, HDAC inhibitors could play a key role in the pathogenesis of neurodegenerative disease in some cases, while in others, they may act as feasible therapeutics for neurodegenerative disease. In general, HDACi have been reported to generate neuroprotective actions. Potential explanations for neuroprotection involve multiple mechanisms of action, which may include but are not limited to: (1) promoting the release of neurotrophic factors, (2) activating kinase pathways, (3) inhibiting extrinsic/intrinsic apoptotic pathways, and (4) suppressing microglia-mediated inflammation [294]. Certain HDACi have been reported to stimulate neurogenesis in pathological conditions [295]. Given that HDACs also have nonhistone substrates in addition to histones, it is possible that more than one pathway contributes to the beneficial effects of HDACi.

Although clinical evidence shows that most HDACi have an acceptable toxicity profile, they lack specificity and may show substantial adverse effects. Due to limitations associated with pan-HDAC inhibitors, the development of isoform-specific HDAC inhibitors would lead to fewer side effects and more selective inhibition. In addition, to enhance the therapeutic efficacy of HDACis, combination therapy might also be an important direction in the future.

### Histone methylation or demethylation modulators

A mounting number of studies suggest that histone methylation may have a crucial role in the pathophysiology of many neurodegenerative diseases. Aberrant histone methylations, such as elevated levels of H3K9me2 [177, 296, 297] and H3K4me3 [298, 299] (a mark of active gene transcription), decreased methylation of H2B K108 and H4 R55 [176], and aberrant intracellular localization of H3K4me3 [300] have been observed in multiple AD/PD models and in the cortex of AD/PD patients, suggesting that restoring histone methylation homeostasis could be a potential therapeutic strategy to treat these neurodegenerative diseases. The histone methyltransferases EMT1 (G9a)/EMT2 (GLP) are responsible for the methylation of H3K9. BIX-01294 (BIX) is a potent inhibitor of histone methyltransferase G9a, which induces demethylation of H3K9 [301]. BIX01294 [302] and another more specific G9a/GLP inhibitor, UNC0642 [303], have been used to restore H3K9me2 levels as well as cognitive functioning in AD mouse models [177]. However, neither of these inhibitors affects AD-related pathologies, suggesting that to improve cognition, it may not be necessary to remove Aβ plaques or reduce tau phosphorylation in AD patients [304]. Unfortunately, these compounds have not yet entered clinical trials.

HDMs mainly include the LSD1 and JmjC families. With the participation of FAD, LSD1 not only specifically removes the dimethyl and monomethyl modification of H3K4 in vitro but can also mediate H3K9me demethylation in vivo [305]. Among the inhibitors of LSD1, some are used to treat neurodegenerative diseases. For example, vafidemastat (ORY-2001), a specific inhibitor of LSD1, has shown potential to slow cognitive impairment and improve memory in AD patients and mice model [306]. This compound is now in a phase II study to assess its safety, tolerability and preliminary efficacy in mild to moderate AD patients (NCT03867253). Significant H3K27me3 enrichment is observed in the brains of PD patients. Recently, GSK-J4, a potent inhibitor of histone demethylase (KDM6A/B and KDM5B/C) with the ability to cross the blood‒brain barrier, has been shown to have strong therapeutic potential for PD. It is reported to rescue the H3K4me3 and H3K27me3 levels in a cellular PD model and rescues dopaminergic neuron loss and motor defects in a PD rat model [307].

In addition, histone methylation-mediated gene regulation has been recognized to be essential in the pathophysiology of various neurodegenerative diseases, such as the α-synuclein coding gene SNCA in PD and C9ORF72 in ALS. However, how specific histone methylation of the genes regulates gene expression needs further study.

### HAT activators

Accumulating evidence implicates the important functions of CBP and p300 in the brain. Unfortunately, studies based on their direct pharmacological activation are still missing due to the lack of cell-permeable activators. A previous study shows that TTK21, a small molecule activator of CBP/p300 conjugated to glucose-based carbon nanospheres (CSPs), can cross the blood‒brain barrier and reach different parts of the brain without causing toxicity [308]. CSP-TTK21 acetylates histones in the hippocampus and frontal cortex to promote newborn neuron development in the dentate subfine particle area. Moreover, although CBP-/-p300 activation does not improve the retention of recent memory, it will significantly extend the duration of memory, which has a beneficial effect on adult neurogenesis and long-term memory brain function [22]. This report provides the first evidence that CBP/p300-mediated activation of histone acetylation in the brain will promote the treatment of brain diseases.

The first study investigating the potential of HAT activators is conducted in a cellular model of PD [309]. CTPB (N-(4-chloro-3-trifluoromethyl-phenyl)-2-ethoxy-6-pentadecylbenzamide), a benzamide and an effective p300 HAT activator, has been demonstrated to have neurotrophic effects in SH-SY5Y neuronal cells. This compound promotes the survival and neurite outgrowth of SH-SY5Y cells by acting on p300/CBP and protects these cells from neurotoxin 6-hydroxydopamine-induced cell death [309]. Inhibitors related to HAT are still in the research phase, and none has entered the clinic yet. The progress of inhibitors related to P300 in the past ten years can be referred to in the review [310], and related to the MYST family can be referred to in this review [311]. Capsaicin (8-methyl-N-vanillyl-trans 6-nonenamide), an agonist of transient receptor potential vanilloid 1 (TRPV1), is found to cause HDAC2 upregulation in the mouse hippocampus, which further reduces the expression of genes related to synaptic plasticity and affects the emotional and neuroplasticity of the brain [312]. Therefore, this study supports the view that capsaicin induces chromatin remodeling with HDAC2 as a molecular link, thus impairing neuron maturation and synaptic plasticity in the hippocampus.

### Bromodomain inhibitors (BRDi)

BRD inhibitors, which compete for binding to acetylated histones, have emerged as promising targets for the treatment of a number of brain diseases. Among the pan-BET family inhibitors, one of the most notable examples is JQ1. It functions as a potent and cell permeable pan-BET family (BRD2, BRD3, BRD4, BRDT) inhibitor. Studies of the therapeutic potential of JQ-1 in multiple mouse models of AD have led to conflicting reports [313, 314]. Using 3 × Tg mice, Magistri et al. find that although JQ1 treatment reduces neuroinflammation and tau phosphorylation in the hippocampus and frontal cortex of AD mice, it shows no beneficial effects on animal cognition [314]. In contrast, Benito et al. report that intraperitoneal administration of JQ1 enhances cognitive performance and long-term potentiation in both WT and APP/PS1-21 AD mice [313]. The discrepancy might be explained by methodological differences such as mouse strain, age, and different experimental paradigms. Therefore, more research is needed to test the effects of BET inhibitors on mouse cognition and to understand how the modulation of BET reader proteins impacts memory function.

Apart from AD, the effectiveness of JQ1 in other neurodegenerative diseases has also been explored. For example, chronic JQ1 administration is found to suppress the induction of levodopa-induced dyskinesia in rats, an animal model for PD [315]. Several BRDi, such as IBET-151, JQ1, and EP72, are shown to increase the expression of expanded C9ORF72 alleles in ALS patient fibroblasts, lymphocytes and reprogrammed motor neurons, conferring therapeutic value for ALS, although further in vivo studies are needed to confirm this [316].

RVX-208, a BD2-selective BET inhibitor, is demonstrated to be safe and well tolerated in a phase Ib/IIa study (NCT01058018). An exploratory phase Ia trial is conducted to evaluate RVX 208 for the treatment of AD. Despite only 24 study subjects, it has demonstrated a 12–14% increase in plasma levels of Aβ_40_ in patients [317], supporting the hypothesis that RVX-208 can augment Aβ_40_ transport from the brain and showing its therapeutic potential for the treatment of AD.

Together, these studies support the view that BET proteins play important roles in many distinct neurodegenerative diseases and that modulation of BET activity may hold therapeutic promise in the management of these disorders.

## Conclusions

Chromatin remodeling is an absolute requirement for gene expression and is thus a vital process in regulating many fundamental biological pathways. In addition, chromatin can be regulated by several processes. These mechanisms function individually and in concert to modulate gene expression. Although tremendous progress has been made in the field of chromatin regulation over the past decade, the mechanisms of the remodeling process are far from being understood and will constitute a fascinating area of research in the future. Particularly, new techniques, such as RNA sequencing (RNA-seq), assays for transposase accessible chromatin using sequencing (ATAC-seq) and others, would prompt important discoveries of chromatin modifications and functions as well as mutations in genes involved in chromatin regulation at a genome-wide level. On the other hand, although previous biochemical and structural studies are informative for the chromatin remodeling mechanisms, detailed studies are needed to understand how remodelers recognize and interact with their targets and the underlying structural basis of chromatin remodeling. New probing techniques such as chromosome conformation capture (3C)^90^, cryo-electron microscopy and quantitative mass spectrometry will be needed to answer such questions. Applying these state-of-the-art techniques would provide new insight into how chromatin remodeling functions during neurodevelopment in health or disease.

Mounting evidence has shown that many neurodegenerative diseases are linked to chromatin deregulation, thus sparking significant efforts to develop new small molecules targeting chromatin remodeling. An increasing number of small molecule inhibitors against a variety of epigenetic regulators have proven effective in the treatment of various neurodegenerative diseases. However, there are still many challenges to overcome. For instance, one major disadvantage of these drugs is their broad specificity and potentially adverse side effects. Furthermore, how can the resistance abilities and blood‒brain barrier penetration of some of these epigenetic drugs be improved? What are the mechanisms of certain inhibitors that cause clinical effects? Only with an improved understanding of the mode of action of these inhibitors can we develop selective, safe and efficacious therapeutic epigenetic drugs. For AD, PD, HD, and ALS, the transfer of promising therapeutic approaches into clinical applications will depend on early-stage treatment. In this regard, compounds that are effectively able to restore distinct aspects of adult neurogenesis might be of specific interest for future clinical studies. Specifically, improving neuropsychiatric and severely disabling symptoms is an urgent need and will have a major impact on the quality of life for all patients suffering from these devastating disorders.

## Data Availability

Not applicable.

## Abbreviations

5hmC: 5-hydroxymethylcytosine
5mC: 5-methylcytosine
8-oxoG: 8-oxoguanine
ABHA: Azelaic bis-hydroxamic acid
AD: Alzheimer’s disease
ALS: Amyotrophic lateral sclerosis
AP: Apical progenitors
ASD: Autism spectrum disorder
BAF: BRG1/BRM-associated factor
BET: Bromodomain and extraterminal domain
BRD: Bromodomain
BRDi: Bromodomain inhibitors
BRK: Brahma and Kismet
C9orf72: Chromosome 9 open reading frame 72
CAF: Chromatin assembly factor
CHD: Chromodomain-helicase DNA-binding protein
CHRAC: Chromatin accessibility complex
CNS: Central nervous system
CRCs: Chromatin remodeling complexes
CTCL: Cutaneous T-cell lymphoma
DG: Dentate gyrus
DNMT: DNA methyltransferase
ESC: Embryonic stem cell
EZH2: Enhancer of Zeste Homologs 2
FA: Friedreich's ataxia
FDA: Food and Drug Administration
FTD: Frontotemporal dementia
FUS: Fused in sarcoma
H3K27me3: H3 lysine 27 trimethylation
H3K36me3: H3 lysine 36 trimethylation
H3K4me3: H3 lysine 4 trimethylation
HAT: Histone acetyltransferase
HD: Huntington’s disease
HDAC: Histone deacetylase
HDACi: HDAC inhibitors
HDM: Histone demethylase
HMT: Histone methyltransferase
HSS: HAND-SANT-SLIDE
INO80: Inositol-requiring 80
IPC: Intermediate progenitor cell
ISWI: Imitation switch
JmjC: Jumonji C
LTP: Long-term synaptic plasticity
NAM: Nicotinamide
NB: Neuroblast
nBAF: Neuron-specific BAF
ncBAF: Noncanonical BAF
NSPC: Neural stem/progenitor cell
NuRD: Nucleosome remodeling and deacetylation
NURF: Nucleosome-remodeling factor
OGG1: 8-OxoGDNA glycosylase 1
OL: Oligodendrocyte
OPC: Oligodendrocyte precursor cell
PBAF: Polybromo-associated BAF
PBRM1: Polybromo-1
PD: Parkinson’s disease
PDE-5: Phosphodiesterase-5
PET: Positron emission tomography
PHD: Plant-homeodomain
PHF2: PHD finger protein 2
PN: Pyramidal neuron
PNS: Peripheral nervous system
polyQ diseases: Polyglutamine diseases
PP1: Protein phosphatase 1
PRC: Polycomb repressive complex
PTCL: Peripheral T-cell lymphoma
PTMs: Posttranslational modifications
qNSC: Quiescent NSPC
REST: Repressor element-1-silencing transcription
RGL: Radial glia-like
RNAPII: RNA polymerase II
RVT: Resveratrol
SBMA: Spinal and bulbar muscular atrophy
SC: Schwann cell
SGZ: Subgranular zone
SMA: Spinal muscular atrophy
SNIBCPS: Snijders Blok-Campeau syndrome
SOD1: Superoxide dismutase 1
SVZ: Subventricular zone
SWI/SNF: Switch/sucrose nonfermentable
TAP: Transient amplifying progenitors
TDP-43: TAR DNA binding protein 43
TET: Ten-eleven translocation
TRPV1: Transient receptor potential vanilloid 1
TRRAP: Transformation/transcription domain-associated protein
TSA: Trichostatin A
VPA: Valproic acid
YY1AP1: YY1 associated protein 1
